# Rapid detection of avian leukosis virus subgroup J by cross-priming amplification

**DOI:** 10.1038/s41598-021-90479-x

**Published:** 2021-05-26

**Authors:** Yong Xiang, Lizhen Li, Peng Liu, Ling Yan, Zeng Jiang, Yun Yu, Yu Li, Xiaoyan Chen, Weisheng Cao

**Affiliations:** 1 College of Veterinary Medicine, South China Agricultural University, No.483 Wushan Road, Tianhe District, Guangzhou, 510642 People’s Republic of China; 2 Key Laboratory of Zoonosis Prevention and Control of Guangdong Province, South China Agricultural University, Guangzhou, 510642 People’s Republic of China; 3 Guangdong Laboratory for Lingnan Modern Agriculture, South China Agricultural University, Guangzhou, 510642 People’s Republic of China; 4 National and Regional Joint Engineering Laboratory for Medicament of Zoonosis Prevention and Control, South China Agricultural University, Guangzhou, 510642 People’s Republic of China; 5 South China Collaborative Innovation Centre for Prevention and Control of Poultry Infectious Diseases and Safety of Poultry Products, South China Agricultural University, Guangzhou, 510642 People’s Republic of China

**Keywords:** Biological techniques, Infectious-disease diagnostics

## Abstract

Avian leukosis virus subgroup J (ALV-J) causes oncogenic disease in chickens in China, resulting in great harm to poultry production, and remains widespread in China. Herein, we employed a cross-priming amplification (CPA) approach and a nucleic acid detection device to establish a visual rapid detection method for ALV-J. The sensitivity of CPA, polymerase chain reaction (PCR) and real-time PCR (RT-PCR) was compared, and the three methods were used to detect ALV-J in the cell cultures which inoculated with clinical plasma. The result showed when the amplification reaction was carried out at 60 °C for just 60 min, the sensitivity of CPA was 10 times higher than conventional PCR, with high specificity, which was comparable with RT-PCR, based on detection of 123 cell cultures which inoculated with clinical plasma, the coincidence rate with real-time PCR was 97.3% (71/73). CPA detection of ALV-J does not require an expensive PCR instrument; a simple water bath or incubator is sufficient for complete DNA amplification, and the closed nucleic acid detection device avoids aerosol pollution, making judgment of results more intuitive and objective. The CPA assay would be a promising simple, rapid and sensitive method for identification of ALV-J.

## Introduction

Avian Leukosis (AL) is an infectious tumour disease affecting poultry caused by avian leukosis viruses (ALVs) belonging to the *Alpharetrovirus* genus of the family *Retroviridae*^1^. Chickens infected with ALVs can suffer from decreased performance and immunosuppression, leading to tumours and even death^2^, it is one of the most important diseases endangering the healthy development of the poultry industry in China^3^. Based on the envelope glycoprotein (gp85) associated with subgroup specificity, ALV in naturally infected flocks can be divided into seven subgroups (A, B, C, D, E, J and K)^4^, among which endogenous nonpathogenic subgroup E viruses with little to no pathogenicity are present in nearly all chicken lines, while the others are exogenous and pathogenic^5,6^. Subgroup A and B viruses mainly cause lymphocytic leukaemia and myeloid leukosis in field flocks^7,8^, while flocks naturally infected with subgroup C and D are rarely observed in the field^9,10^.

Avian leukosis virus subgroup J (ALV-J) was first isolated and identified in broiler chickens in 1988^11^. Chickens infected with ALV-J can suffer from medulloblastoma and decreased production performance, causing huge economic losses to the poultry breeding industry across the world^12^. ALV-J was originally thought only to infect meat-type chickens^13^, but its host range is now known to include layer chickens and local broiler breeders in most parts of China^14,15^, and the disease has even spread to waterfowl and wild birds^16^. Most large foreign breeder companies had eradicated classic exogenous ALVs by the end of the 1980s, but ALV-J remains widespread in China^17^.

Effective vaccines and drugs have not yet been reported for AL. Obtaining a population of breeding poultry without exogenous ALV through purification is therefore the main method for disease prevention and control, for which virus detection is crucial. A variety of methods for detecting and differentially diagnosing ALV have been established in various regions of the world, mainly involving virus isolation and identification^18^, pathological diagnosis^19^, serological detection methods such as enzyme-linked immunosorbent assay (ELISA)^20^, and PCR-based molecular biological detection^21^. However, existing detection methods have some limitations and shortcomings. For example, traditional virus isolation and identification is too time-consuming to meet the requirements of rapid diagnosis, and PCR and real-time PCR (RT-PCR) require expensive instruments and equipment not conducive to fieldwork. Additionally, while the loop-mediated isothermal amplification (LAMP) detection method does not require specific instruments or equipment^22^, it is highly prone to false positives, weak positive results tend to be inaccurate, and contamination by aerosols can occur easily.

Cross-priming amplification (CPA) is a rapid detection method developed in recent years^23–25^. The CPA utilizes multiple primers and probes to amply nucleotide sequence isothermally. By labelling one primer with biotin, another primer with FITC, then the amplified products were recognized by anti-biotin and anti-FITC monoclonal antibodies on the test line, where gold nanoparticles (AuNPs) were fixed, CPA amplified products could be visualized in nucleic acid test strip^26^. It benefits from high specificity and sensitivity, does not require expensive instruments, the operation process is relatively simple, amplification is rapid, and it can be used with disposable nucleic acid detection strip in closed tubes, thereby avoiding aerosol pollution, making results more intuitive and objective^27^. This method has been applied to the diagnosis of infectious diseases and the detection of multiple pathogens^24,28^. In the present study, we developed a CPA assay for ALV-J, which would be a promising rapid user-friendly detection method.

## Results

### Establishment of the basic amplification system

**Figure 1:**
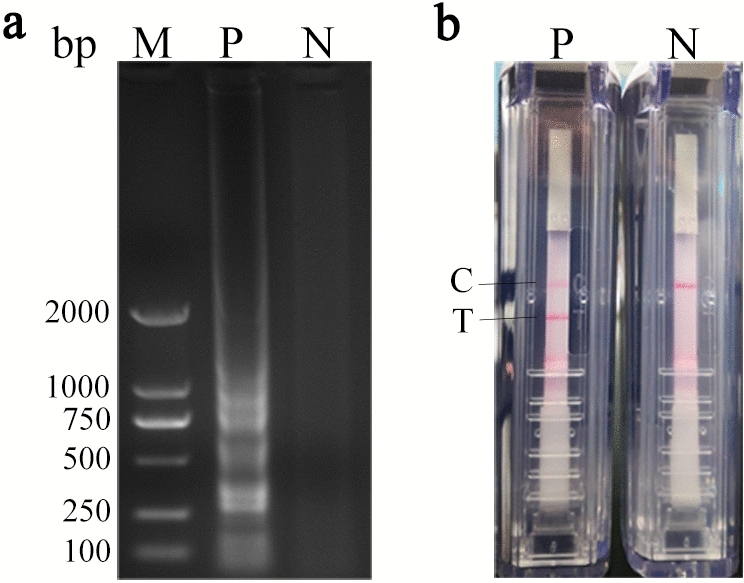
The basic reaction system of ALV-J CPA reaction. (**a**) Agarose gel electrophoresis of test samples; The full-length gel is presented in Supplementary Figure S17. (**b**) Nucleic acid detection strips of test samples. Lane M, DNA markers; P, ALV-J; *N* negative control, *C* control-line, *T* test-line.

When the CPA product was observed under ultraviolet light following electrophoresis, it gave a ladder-like pattern on the agarose gel, result of their characteristic structure, but the negative control was not (Fig. 1a). The results of Disposable Nucleic Acid Detection Strip showed that the positive control containing the recombinant plasmid as template yield red bands on both the control line (C) and the test line (T), while the negative control group only yielded the C line (Fig. 1b), which suggests that the basic CPA reaction system could amplify ALV-J effectively.

### Optimization of the reaction system and conditions

**Figure 2:**
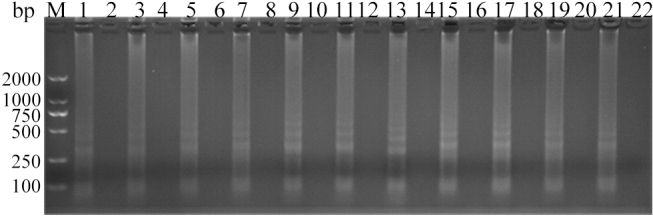
Analysis of ALV-J CPA at different concentration of primers (Table 1) by agarose gel electrophoresis. Lane M, DNA marker; 1, 3, 5, 7, 9, 11, 13, 15, 17, 19, 21, were primers groups 1–11 (Table 1) respectively; 2, 4, 6, 8, 10, 12, 14, 16, 18, 20 and 22 were negative controls for the corresponding primers groups. The full-length gel is presented in Supplementary Figure S18.

**Figure 3:**
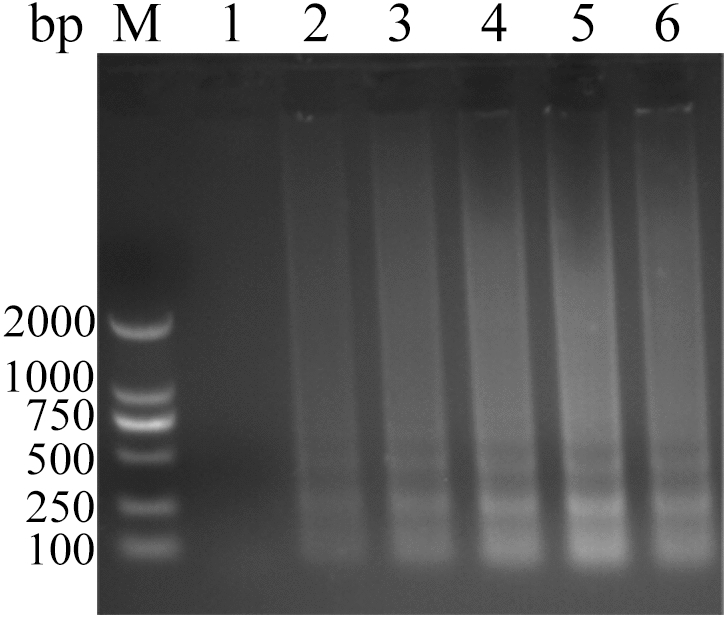
Analysis of ALV-J CPA at different concentration of Mg^2+^ by agarose gel electrophoresis. Lane M, DNA marker; 1, 0 mmol L^−1^; 2, 1 mmol L^−1^; 3, 2 mmol L^−1^; 4, 3 mmol L^−1^; 5, 4 mmol L^−1^; 6, 5 mmol L^−1^. The full-length gel is presented in Supplementary Figure S19.

**Figure 4:**
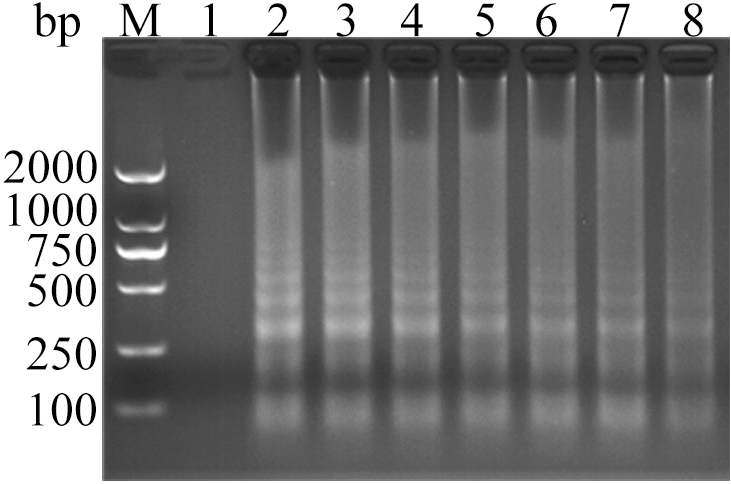
Analysis of ALV-J CPA at different concentration of Betaine by agarose gel electrophoresis. Lane M, DNA marker; 1, 0 mol L^−1^; 2, 0.2 mol L^−1^; 3, 0.4 mol L^−1^; 4, 0.6 mol L^−1^; 5, 0.8 mol L^−1^; 6, 1.0 mol L^−1^; 7, 1.2 mol L^−1^; 8, 1.4 mol L^−1^. The full-length gel is presented in Supplementary Figure S20.

**Figure 5:**
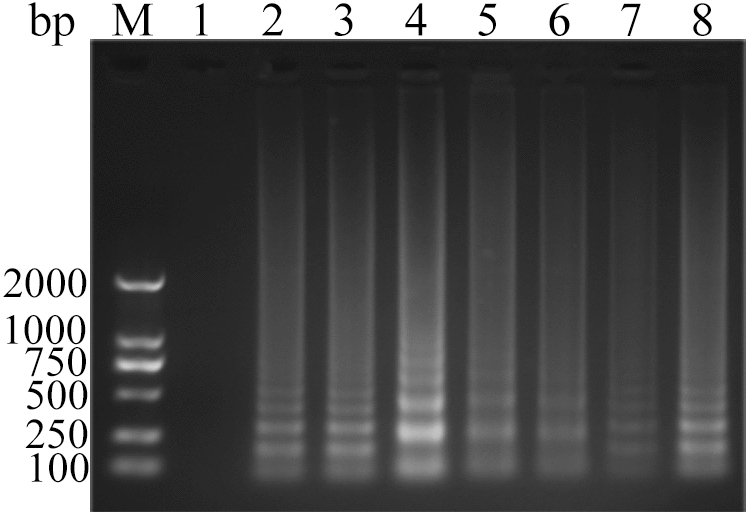
Analysis of ALV-J CPA at different concentration of dNTPs by agarose gel electrophoresis. Lane M, DNA marker; 1, 0 mmol L^−1^; 2, 0.2 mmol L^−1^; 3, 0.4 mmol L^−1^; 4, 0.6 mmol L^−1^; 5, 0.8 mmol L^−1^; 6, 1.0 mmol L^−1^; 7, 1.2 mmol L^−1^; 8, 1.4 mmol L^−1^. The full-length gel is presented in Supplementary Figure S21.

**Figure 6:**
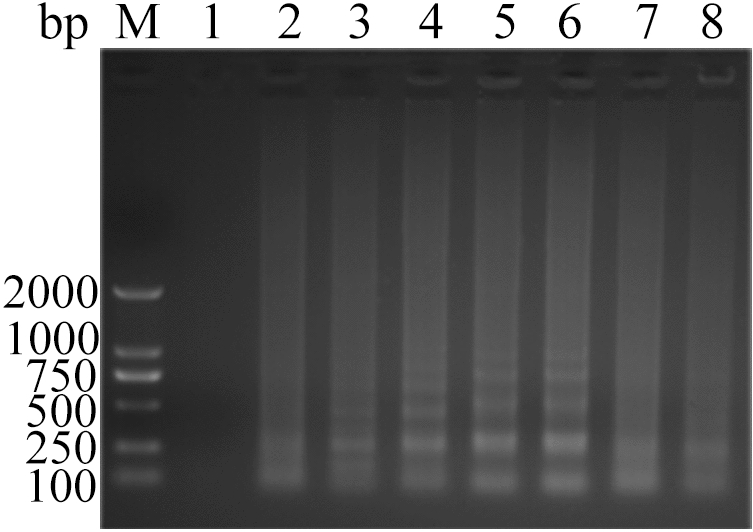
Analysis of ALV-J CPA at different units of *Bst* DNA polymerase (8 units μL^−1^) by agarose gel electrophoresis. Lane M, DNA markers; 1, 0 units μL^−1^; 2, 0.064 units μL^−1^; 3, 0.128 units μL^−1^; 4, 0.192 units μL^−1^; 5, 0.256 units μL^−1^; 6, 0.32 units μL^−1^; 7, 0.48 units μL^−1^; 8, 0.64 units μL^−1^. The full-length gel is presented in Supplementary Figure S22.

**Figure 7:**
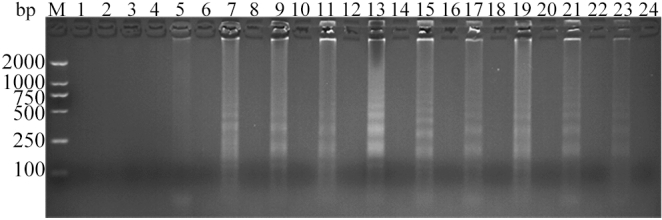
Analysis of ALV-J CPA at different temperatures by agarose gel electrophoresis. Lane M, DNA marker; 1, 54 °C; 3, 55 °C; 5, 56 °C; 7, 57 °C; 9, 58 °C; 11, 59 °C; 13, 60 °C; 15, 61 °C; 17, 62 °C; 19, 63 °C; 21, 64 °C; 23, 65 °C; 2, 4, 6, 8, 10, 12, 14, 16, 18, 20, 22 and 24 were negative controls for the corresponding temperatures. The full-length gel is presented in Supplementary Figure S23.

The optimal primers concentration was primer group 7 (Fig. 2, Table 1), based on strips that were clearer and brighter. Optimal concentrations for Mg^2+^, betaine, dNTPs and *Bst* DNA polymerase in the amplification system were 4 mmol L^−1^ for Mg^2+^ (Fig. 3), 0.2 mol L^−1^ for Betaine (Fig. 4), 0.6 mmol L^−1^ for dNTPs (Fig. 5), and 0.32 units μL^−1^ for *Bst* DNA polymerase (Fig. 6). The reaction system was tested at 54, 55, 56, 57, 58, 59, 60, 61, 62, 63, 64 and 65 °C for 60 min, and the optimal temperature was 60 °C (Fig. 7) (Supplementary Fig. S11–Fig. S12).

**Table 1:** Different primer concentration combinations.

Primer groups	Working concentration (µmol·L^−1^)				
	F3	B3	F2	F1	CPR
1	0.4	0.4	0.6	0.6	0.8
2	0.4	0.4	0.8	0.8	1.0
3	0.4	0.4	0.6	0.6	1.0
4	0.4	0.4	0.6	0.6	1.2
5	0.2	0.2	0.8	0.8	1.2
6	0.2	0.2	0.4	0.4	0.6
7	0.2	0.2	0.8	0.8	1.0
8	0.2	0.2	0.6	0.6	1.0
9	0.6	0.6	0.8	0.8	1.0
10	0.6	0.6	1.0	1.0	1.2
11	0.6	0.6	0.8	0.8	1.2

### Optimization of the ALV-J CPA assay reaction time

**Figure 8:**
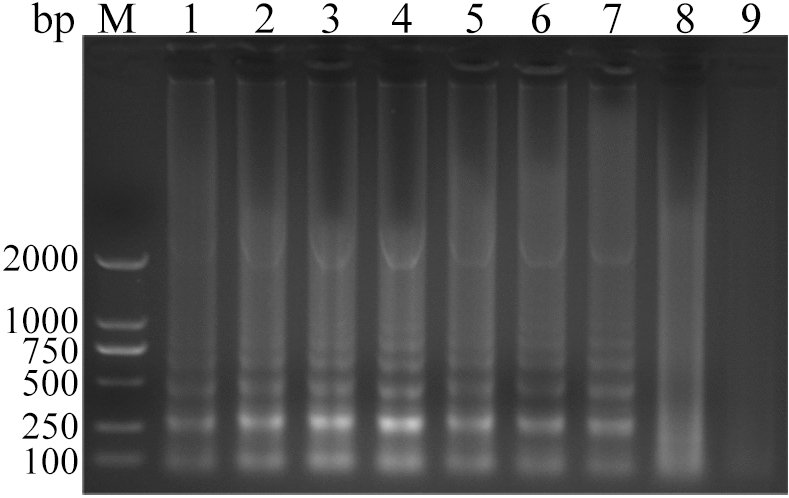
Analysis of ALV-J CPA with different reaction times by agarose gel electrophoresis. Lane M, DNA marker; 1, 15 min; 2, 30 min; 3, 45 min; 4, 60 min; 5, 75 min; 6, 90 min; 7, 105 min; 8, 120 min; 9, Negative control amplification for 120 min. The full-length gel is presented in Supplementary Figure S24.

The ALV-J CPA reaction system was amplified at 60 °C for 15 min, 30 min, 45 min, 60 min, 75 min, 90 min, 105 min and 120 min respectively. The results showed that the amplification products reached the maximum at 60 min. However, when the reaction time exceeded 60 min, the reaction effect decreased. Therefore, 60 min was optimal (Fig. 8) (Supplementary Fig. S13- Fig. S14).

### Specificity of the ALV-J CPA assay

**Figure 9:**
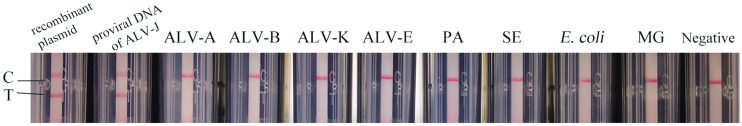
Analysis of the specificity of the ALV-J CPA method. *PA* *Pseudomonas aeruginosa,* *SE* *Salmonella enteritis*, *MG* *Mycoplasma gallisepticum*, *C* control-line, *T* test-line.

Specific experiments were carried out using the optimal reaction system and conditions for ALV-J CPA. The results showed that only recombinant plasmid standards and ALV-J cDNA yielded positive test line and control line results, while ALV-A, ALV-B, ALV-K, ALV-E, PA, *E.coli,* SE and MG yielded only positive control line results (Fig. 9), indicating that the ALV-J CPA detection method is highly specific.

### Evaluation of the sensitivity of the CPA assay

**Figure 10:**
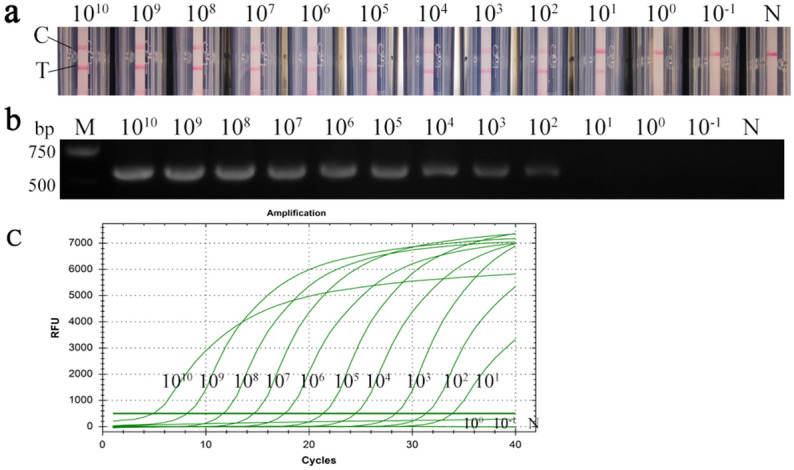
Comparison of sensitivity between the CPA, PCR and RT-PCR of ALV-J detect methods based on recombinant plasmid standards at 1.86 × 10^–1^ ~ 1.86 × 10^10^ copies μL^−1^ (a representative image of the three independent) (**a**) Sensitivity of CPA based on a nucleic acid detection device; (**b**) Sensitivity of conventional PCR; The full-length gel is presented in Supplementary Figure S25. (**c**) Sensitivity of RT-PCR for ALV-J. C, control-line; T, test-line; N, negative control.

Sensitivity tests were carried out using the optimal reaction system for CPA. The results showed that when the plasmid DNA copy number was between 1.86 × 10^1^ and 1.86 × 10^10^ copies μL^−1^, identification was possible with nucleic acid test strips. By contrast, only the control line was appeared at 1.86 × 10^–1^ copies μL^−1^ and 1.86 copies μL^−1^, hence the detection limit of the ALV-J CPA method was 1.86 × 10^1^ copies (Fig. 10a). The results of conventional PCR amplification showed that the target band was amplified only at 1.86 × 10^2^–1.18 × 10^10^ copies μL^−1^, equating to a detection limit of 1.86 × 10^2^ copies (Fig. 10b). Thus, the sensitivity of ALV-J CPA was up to 10 times higher than that of conventional PCR. The results of RT-PCR amplification showed that when the plasmid DNA copy number was between 1.86 × 10^1^ and 1.86 × 10^10^ copies μL^−1^, the cycle number was below 35 and consider as positive (Fig. 10c) (Supplementary Fig. S15–Fig. S16). It shows that the ALV-J CPA method is as sensitive as RT-PCR.

### Detection of ALV-J in cell cultures inoculated with clinical plasma samples

A total of 48 cell cultures of ALV p27-positive which inoculated with clinical plasma (S/P > 0.2) and 75 cell cultures inoculated with plasma with 0.1 ≤ S/P ≤ 0.2 based on ELISA were tested for ALV-J using the CPA method, conventional PCR, and RT-PCR. The results showed that, among the 48 samples with S/P > 0.2, the positive rates for CPA, real-time PCR and PCR were 54.2% (26/48), 54.2% (26/48) and 52.1% (25/48), respectively. The samples which showed ALV-J positive for CPA and RT-PCR were consistent, and the 25 samples that tested positive by PCR were included in the 26 samples tested positive for CPA. Detailed results are shown in Table 2 and Supplementary Table S1. Among the 75 samples with 0.1 ≤ S/P ≤ 0.2, the positive rates for CPA, RT-PCR and PCR were 60.0% (45/75), 62.6% (47/75) and 38.7% (29/75), respectively. And the 45 samples that tested ALV-J positive by CPA were included in the 47 samples tested positive for RT-PCR. Detailed results are shown in Table 3 and Supplementary Table S2. The sensitivity of the ALV-J CPA assay was higher than that of conventional PCR, which was comparable with RT-PCR. Among a total of 123 cell cultures which inoculated with clinical plasma, 71 samples tested positive for CPA and 73 samples tested positive for RT-PCR, and the 71 samples tested positive for CPA were included in the 73 samples tested positive for RT-PCR, so the coincidence rate of the two methods was 97.3% (71/73).

**Table 2:** Detection results for cell cultures inoculated with clinical plasma with S/P > 0.2.

Detected number	RT-PCR		PCR		Total
	Positive	Negative	Positive	Negative	
**CPA**					
Positive	26	0	25	1	26
Negative	0	22	0	22	22
Total	26	22	25	23	48

**Table 3:** Detection results for cell cultures inoculated with clinical plasma with 0.1 ≤ S/P ≤ 0.2.

Detected number	RT-PCR		PCR		Total
	Positive	Negative	Positive	Negative	
**CPA**					
Positive	45	0	29	16	45
Negative	2	28	0	30	30
Total	47	28	29	46	75

## Discussion

ALV-J infection currently causes great economic losses in China^17^, and it presence is associated with a variety of tumour types including myeloma, renal tumours and hemangioma^19,29^. The high incidence of tumours reduces production, and virus elimination increases production costs. In order to meet the need for rapid diagnosis, it is necessary to develop a simple, rapid and sensitive diagnostic method for detecting ALV-J. The isolation and identification of viruses by cell culture is expensive and time-consuming. Conventional PCR and real-time PCR is an established method for detecting ALV-J^30^, but it requires high-precision instruments, skilled operators, and the amplification time is 3 to 4 h. Additionally, it is difficult to adapt this method for rapid detection of ALV-J in the field. ELISA is a method that can potentially solve this problem since it can detect whether a sample contains the ALV p27 antigen, but it cannot be used for the identification of ALV subgroups, nor can it distinguish exogenous from endogenous ALV.

In recent years, researchers have paid increasing attention to new detection methods based on molecular biology. CPA is a gene amplification approach that, compared with traditional PCR, pathogen separation and similar methods, has many advantages, including being faster and more accurate. One of the most important features of CPA is its high amplification efficiency and sensitivity. Even a small number of templates can produce a large number of amplification products^31^. The application of CPA technology not only overcomes the dependence on PCR and RT-PCR instruments, but also affords an operational procedure that is simple and accurate, and interpretation of results is more objective, convenient and intuitive than is the case for loop-mediated isothermal amplification (LAMP), since it avoids subjectivity^22^. CPA technology facilitates molecular biology-based detection by non-specialist laboratories and economically underdeveloped areas, which is of great significance for the scientific research of nucleic acids and viral diseases^32^.

In the process of developing a successful CPA assay, we found that primer design was crucial^25^. For accurate detection, the primers play a vital role in ensuring specificity and sensitivity in this assay and their design is one of the difficulties of CPA. Because the CPA assay makes use of five primers to recognize five distinct regions on the target DNA sequence, which makes the CPA assay more specific than conventional PCR using two primers^26^. The presence of multiple primers in the same tube can result in non-specific amplification leading to false positives and misleading detection results^33^. Before determining the final ALV-J CPA primers, we had made a lot of preliminary experiments to screened multiple primer sets and eliminated those giving non-specific amplification products.

In the present study, a rapid CPA detection method for ALV-J was established. Optimal reaction conditions were 60 °C for 60 min, and the sensitivity was 10 times that of conventional PCR. The assay proved negative for other ALV subgroups, and other virus, and bacteria. Our laboratory has long been committed to the detection and purification of avian leukosis. The clinical plasma samples were inoculated into DF-1 cells and kept for continuous 7–9 days. Then the p27 antigen in the cell supernatant was detected by ELISA kit, and the DNA of cell precipitation was extracted, and then the different subgroup of ALV were identified by PCR or RT-PCR, so as to complete the separation and identification of ALV.

In previous studies, Dai et al. established the RT-PCR method to detect ALV-J, which was 100 times more sensitive than virus isolation and 10 times than conventional PCR. What’s more, ALV-J was also detected in cell cultures incubated with clinical plasma samples, and the results showed that the positive rates of conventional PCR and RT-PCR were higher than that of virus isolation. That is to say, the sensitivity of RT-PCR and conventional PCR is higher than that of virus isolation^30^. And the RT-PCR method of ALV-J used in this study is exactly the one established by Dai et al.

In the process of virus isolation, the ELISA kit for p27 is an important method. The operation is carried out according to the IDEXX kit instructions, and the S/P value is calculated. When S/P > 0.2, it is considered as a positive sample, indicating the presence of exogenous ALV p27 antigen. When S/P ≤ 0.2, it is considered as a negative sample by ELISA method, that is, there is no enough ALV p27 exist according to manufacturer's manual. However, in this continuous process of purification, we found that ALV can be detected by RT-PCR in some cell cultures with 0.1 ≤ S/P ≤ 0.2, and even a few samples can show positive by PCR and isolate the virus, so we think it is weakly positive for p27. This is why in Dai et al. 's study, the positive rate of both conventional PCR and RT-PCR was higher than that of virus isolation. Therefore, the selection in this study is no longer compared with virus isolation, but directly compared with RT-PCR was as convincing. And we collected cell cultures with S/P > 0.2, extracted cell DNA, and used CPA, PCR and RT-PCR to detect ALV-J to verify the reliability of the CPA method established in this study. Secondly, we also collected cell cultures with 0.1 ≤ S/P ≤ 0.2, extracted cell DNA and still used the above three methods to detect ALV-J to verify the sensitivity of the CPA method established in this study.

As a result, among the 48 p27-positive samples (S/P > 0.2), the ALV-J positive samples detected by CPA and RT-PCR were overlapped, while the 25 PCR positive samples were also included in the 26 RT-PCR positive samples. Among the 75 samples of p27 weakly positive (0.1 ≤ S/P ≤ 0.2), the 45 samples that tested ALV-J positive by CPA were included in the 47 samples tested positive for RT-PCR, and the 29 samples that tested ALV-J positive by PCR were included in the 45 samples tested positive for CPA. Therefore, the CPA method is sensitive and reliable as RT-PCR, and the sensitivity is higher than conventional PCR. Since the sensitivity of p27 ELISA was lower than that of PCR, RT-PCR and CPA, there should be no samples tested positive by PCR but negative by RT-PCR or CPA. As long as PCR is positive, then RT-PCR and CPA must be negative, and the results were indeed so.

In summary, the established ALV-J CPA method has significant advantages for the detection of ALV-J. It is convenient to execute, exhibits strong specificity, high sensitivity, and does not require complicated experimental equipment, making it suitable for analysis outdoors and in primary medical units. It is an ideal system for rapid detection of ALV-J with great potential for monitoring this viral disease.

## Methods

### Main experimental materials and reagents

ALV-J (HN06), ALV-A (GD-13), ALV-B (CD08), ALV-K (GDFX0601), ALV-E (HN1301) and *Salmonella enteritis* (SE) were maintained in our laboratory. *Escherichia coli* ATCC15922 and *Pseudomonas aeruginosa* ATCC27853 were obtained from HuanKai Microbial (Guangzhou, China). *Mycoplasma gallisepticum* (MG) S6 was donated by Professor Ding (College of Veterinary Medicine, South China Agricultural University). *Bst* DNA polymerase, 10 × ThermoPol Reaction Buffer, dNTPs and MgSO_4_ were products of New England Biolabs (Beverley, MA, USA). Betaine was acquired from Sigma (St. Louis, MO, USA). Disposable Nucleic Acid Detection Strip were purchased from Ustar (Hangzhou, China). The DNA Extraction Kit, Gel Extraction Kit, and Plasmid Mini Kit were produced from OMEGA (Norcross, GA, USA).

### Primer design

**Figure 11:**
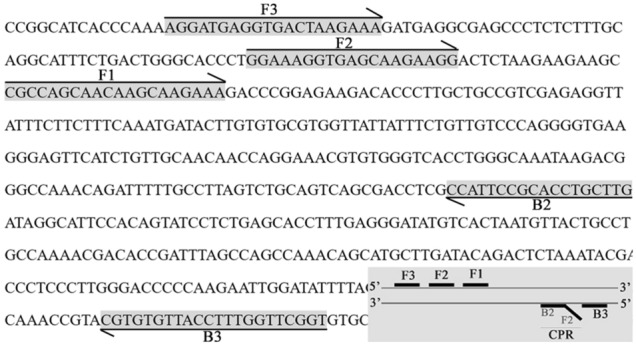
The specific positions and sequence of CPA primers and probes in the genome.

Using ALV-J sequences published in the NCBI database (GeneBank ID: HQ900844.1, HM776937.1, JQ935966.1 and Z46390.1), online software PrimerExpoler (http://primerexplorer.jp/e/), Primer Premier 5.0 and Oligo 7 were used to design CPA primers and probes for the envelope glycoprotein gene of ALV-J. Include two external primers (F3 and B3), one cross-amplification primer named as CPR which consist of two short primers (F2 and B2), but primer B2 does not exist independently in the reaction system, it’s only a part of the sequence of primer CPR. In addition, two probe primers are included in the reaction system, namely F1 (labelled at the 5′ end with FAM) and F2 (labelled at the 5′ end with biotin). The amplified envelope glycoprotein gene fragment was 548 bp. The specific positions and sequence of primers and probes in the genome are shown in Fig. 11 and Table 4. These primers and probes were synthesised by Sangon Biotech (Shanghai, China) and diluted to 10 μmol L^−1^ before use.

**Table 4:** Primer and probe sequences.

Primer	Sequence(5′ − 3′)
F3	AGGATGAGGTGACTAAGAAA
B3	ACCGAACCAAAGGTAACACACG
B2	CAAGCAGGTGCGGAATGG
F2	Biotin-GGAAAGGTGAGCAAGAAGG
F1	FAM-CGCCAGCAACAAGCAAGAAA
CPR	GGAAAGGTGAGCAAGAAGGCAAGCAGGTGCGGAATGG

### DNA extraction and quantification of recombinant plasmid standards

Total DNA was extracted from DF-1 cells (2.5 × 10^6^ cells) infected with ALV-J (2 × 10^3^ TCID_50_) using a DNA Extraction kit (OMEGA, Norcross, GA) according to the manufacturer’s instructions and stored at − 20 °C until use. The PCR amplification system and reaction conditions for the 548 bp target fragment amplified using primer F3 and B3 comprised 10 μL of 2 × Premix r Taq (YEASE, Shanghai, China), 1 μL of primer F3 (10 μmol L^−1^) and B3 (10 μmol L^−1^), 1 μL of total DNA template (100 ng/μL,), and deionised water up to 20 μL. An PCR System (Applied Biosystems, FosterCity, CA) was used for amplification with 1 cycle of 95 °C for 5 min, 40 cycles of 95 °C for 1 min, 55 °C for 1 min and 72 °C for 1 min, 1 cycle of 72 °C 5 min. The PCR product was recovered and cloned into the pMD-18 T vector according to the manufacturer’s instructions (Takara, Japan), recombinants were transformed into *E. coli* DH5α competent cells (Takara, Japan), monoclonal clones were selected on LB plates containing ampicillin, DNA was extracted from positive clones using a Plasmid Mini Kit (OMEGA, Norcross, GA), and sent to Sangon Biotech (Shanghai, China) for DNA sequencing. Plasmid concentration were measured by UV spectroscopy (Thermo, USA ) and the recombinant plasmid copy number was calculated according to the formula: number of copies = (concentration in ng × 6.02 × 10^23^) / (genome length × 10^9^ × 660)^34^, and the samples were stored at − 20 °C until use.Finally, the concentration of the recombinant plasmid standard was measured to be 66 ng μL^−1^, the purity of the plasmid was 1.94. The length of the pMD-18 T vector was 2692 bp, the length of the insert was 548 bp, hence the total number of bases was 3240 bp, substituting it into the formula and calculating the copy number is 1.86 × 10^10^ copies μL^−1^.

### Establishment of the basic amplification system and optimisation of reaction conditions

The basic CPA reaction system was established as described previously^31^. Reactions were performed a 25 μL total volume in 0.2 mL centrifuge tube, which containing 1 × Thermo Pol Buffer, 0.4 mol L^−1^ of Betaine, 0.8 mmol L^−1^ of dNTPs, 4 mmol L^−1^ of Mg^2+^, 8 units of *Bst* DNA polymerase, 0.8 μmol L^−1^ of CPR primer, 0.6 μmol L^−1^ of F1 primer, 0.6 μmol L^−1^ of F2 primer, 0.4 μmol L^−1^ of F3 primer, 0.4 μmol L^−1^ of B3 primer and 2.0 μL of template DNA. The reaction was carried out at 60 °C for 60 min. The products were detected by 2% agarose gel electrophoresis in 1 × TAE with EB staining and the Disposable Nucleic Acid Detection Strip (Ustar, Hangzhou, China) according to the manufacturer’s instructions, when CPA amplification was completed. In brief, the 0.2 mL centrifuge tube after the reaction is embedded in the Disposable Nucleic Acid Detection Strip, then press the handle forcefully, and observe the results after 5–10 min. Recombinant plasmid standards and empty plasmid were used as positive template and negative controls, respectively. The basic reaction system could then be optimised if applicable. As shown in Table 1, different concentrations of primers were optimised, and each combination included a negative control. The reaction system was used to optimise the concentration of betaine, Mg^2+^, dNTPs and *Bst* DNA polymerase by varying each one in turn. Finally, the reaction temperature and time were optimised. All these analyses were repeated three times.

### Specificity and sensitivity of the CPA assay

DNA was extracted from PA, SE, *E. coli*, MG and DF-1 cells infected with ALV-J, ALV-A, ALV-B, ALV-K and ALV-E using an SQ tissue DNA kit (OMEGA) according to the manufacturer’s instructions. And diluted each DNA at the same concentration (50 ng μL^−1^). The specificity of the CPA method was determined using the above nucleic acids as templates. A tenfold dilution series of ALV-J recombinant plasmid DNA was prepared using sterile ddH_2_O, and different copy number of plasmid DNA were amplified by the CPA assay, conventional PCR (F3 and B3 as primes, described above), and RT-PCR^30^. RT-PCR was carried out in a 20 μL reaction volume containing 10 μL 2 × iTaq Universal SYBR Green Supermix (BioRad, Hercules, CA), 1.0 μL each primer (Forward: 5′-TGTGTGCGTGGTTATTATTTC-3′, Reverse: 5′-AATGGCGAGGTCGCTGACTGC-3′) (10 pmol μL^−1^), 1 μL plasmid and 7 μL ddH_2_O. An ABI 7500 Real-time PCR System (Applied Biosystems, FosterCity, CA) was used for amplification with 1 cycle of 95 °C 3 min; 40 cycles of 95 °C for 15 s and 60 °C for 34 s. Fluorescence signals were collected after each amplification step. All these analyses were repeated three times.

### Detection of ALV-J in cell cultures inoculated with clinical plasma samples

During the purification of avian leukosis, the anticoagulant blood of chickens collected from many different poultry farms were sent back to the laboratory, then centrifuged at 4 °C 2000*g* for 6 min, and the plasma (80 μL) was isolated and inoculated into DF-1 cells (2 × 10^5^ cells per well) and kept for continuous 7–9 days. Then the p27 antigen in the cell supernatant was detected by ELISA kit (IDEXX), according to ELISA analysis of p27, the cell cultures of ALV p27-positive (S/P > 0.2) were collected, and the cell DNA was extracted to detect ALV-J by CPA method, routine PCR (F3 and B3 as primers) and RT-PCR^30^, to compare the consistency of the test results of the three methods. Additionally, the cell cultures with 0.1 ≤ S/P ≤ 0.2 which inoculated with clinical plasma were also collected to extracted cell DNA to detect ALV-J by the three methods, and the sensitivity of the three methods was compared. All these analyses were repeated three times. DNA was extracted with DNA Extraction kit (OMEGA, Norcross, GA) according to the manufacturer’s instructions.

### Ethics statement

The plasma samples were from yellow chickens of Guangdong province in China during an ALV epidemiological investigation conducted by our laboratory. And all the chicken plasma sampling procedures were approved by the Animal Care and Use Committee of Guangdong Province, China (2018B110). Our sampling processes were assisted by local authorities and veterinarians. And all experiments were performed in accordance with relevant guidelines and regulations.

## Supplementary Information

Supplementary Information.

## Data Availability

All data generated or analysed during this study are included in this published article (and its Supplementary Information files).
