# Supplementary material for: Rapid detection of avian leukosis virus subgroup J by cross-priming amplification
Source: Sci Rep. 2021 May 26;11:10946. doi: 10.1038/s41598-021-90479-x (PMC8155010; doi:10.1038/s41598-021-90479-x)
Supplement: Supplementary file 1 — Supplementary Information. [file 41598_2021_90479_MOESM1_ESM.pdf]

Supplement Information

Rapid Detection of Avian Leukosis Virus Subgroup J by Cross-priming Amplification

Yong Xiang[1], Lizhen Li[1], Peng Liu[1], Ling Yan[1], Zeng Jiang[1], Yun Yu[1], Yu Li[1], Xiaoyan Chen[1], Weisheng Cao[1,2,3,4,5,*]

Author affiliations:

[1] College of Veterinary Medicine, South China Agricultural University, Guangzhou 510642, P.R.China.

[2] Key Laboratory of Zoonosis Prevention and Control of Guangdong Province, South China Agricultural University, Guangzhou 510642, P.R.China.

[3] Guangdong Laboratory for Lingnan Modern Agriculture, South China Agricultural University, Guangzhou 510642, P.R.China.

[4] National and Regional Joint Engineering Laboratory for Medicament of Zoonosis Prevention and Control, South China Agricultural University, Guangzhou 510642, P.R.China.

[5] South China Collaborative Innovation Centre for Prevention and Control of Poultry Infectious Diseases and Safety of Poultry Products, South China Agricultural University, Guangzhou 510642, P.R.China.

E-mail:

Yong Xiang: xiangyongcq@163.com

Lizhen Li: 2530478390@qq.com

Peng Liu: liupeng121891@163.com

Ling Yan: 624798838@qq.com

Zeng Jiang: 18825075245@163.com

Yun Yu: 1171501813@qq.com

Yu Li: 145bly@163.com

Xiaoyan Chen: 2196174054@qq.com

Weisheng Cao: caoweish@scau.edu.cn

*** Corresponding author:** Weisheng Cao (PhD, Professor)

E-mail: caoweish@scau.edu.cn

Address: College of Veterinary Medicine, South China Agricultural University, No.483 Wushan Road, Tianhe District, Guangzhou, 510642, China

Telephone: 86-20-85282536 (office); **mobile:** 86-13318865369

Contents

Supplementary Figures： ·· 1

The full-length gels used in the main article which were cropped: ········· 9

The full-length gels used in the Supplementary Figures which were cropped: 14

Supplementary Tables： ·· 24

Supplementary Figures：

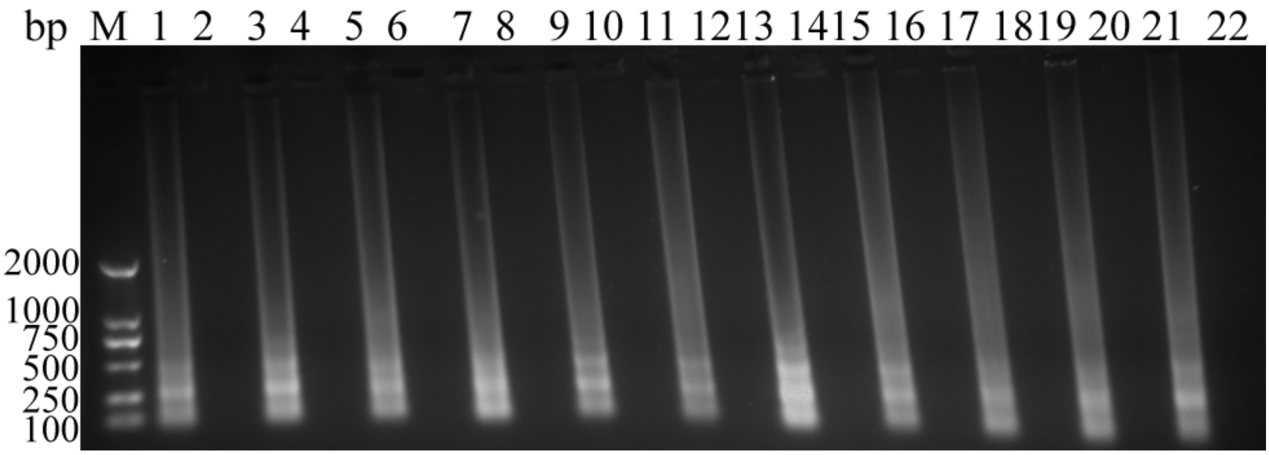

Supplementary Figure S1 Analysis of ALV-J CPA at different concentration of primers (Table 1) by agarose gel electrophoresis (repeated analyze). Lane M, DNA marker; 1, 3, 5, 7, 9, 11, 13, 15, 17, 19, 21, were primers groups 1-11 (Table 1) respectively; 2, 4, 6, 8, 10, 12, 14, 16, 18, 20 and 22 were negative controls for the corresponding primers groups. The full-length gel is presented in Supplementary Figure S26.

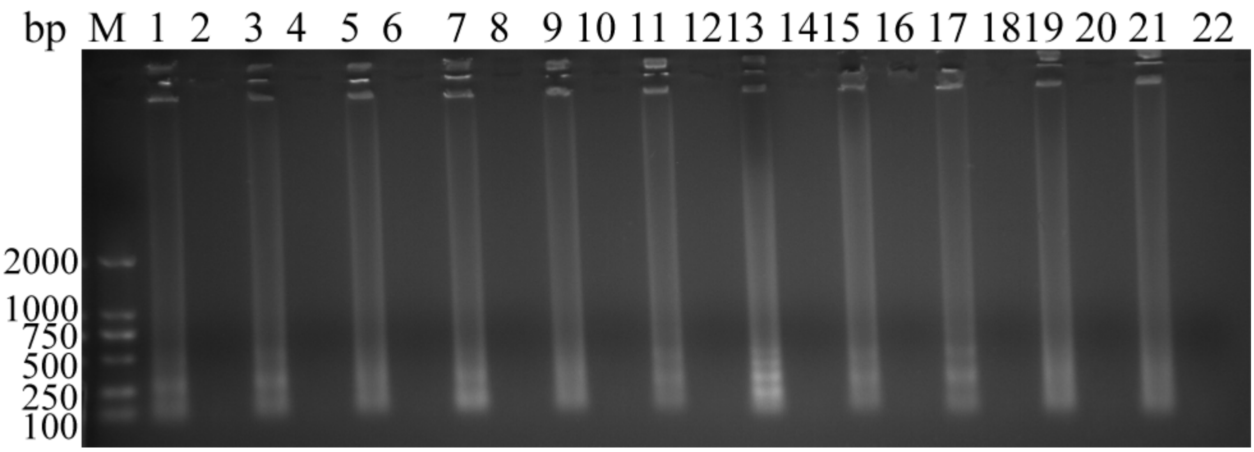

Supplementary Figure S2 Analysis of ALV-J CPA at different concentration of primers (Table 1) by agarose gel electrophoresis (repeated analyze). Lane M, DNA marker; 1, 3, 5, 7, 9, 11, 13, 15, 17, 19, 21, were primers groups 1-11 (Table 1) respectively; 2, 4, 6, 8, 10, 12, 14, 16, 18, 20 and 22 were negative controls for the corresponding primers groups. The full-length gel is presented in Supplementary Figure S27.

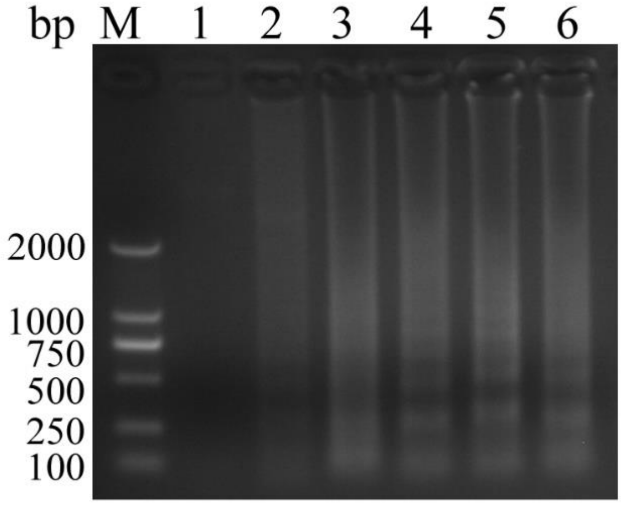

Supplementary Figure S3 Analysis of ALV-J CPA at different concentration of Mg^{2+} by agarose gel electrophoresis (repeated analyze). Lane M, DNA marker; 1, 0 mmol L^{-1}; 2, 1 mmol L^{-1}; 3, 2 mmol L^{-1}; 4, 3 mmol L^{-1}; 5, 4 mmol L^{-1}; 6, 5 mmol L^{-1}. The full-length gel is presented in Supplementary Figure S28.

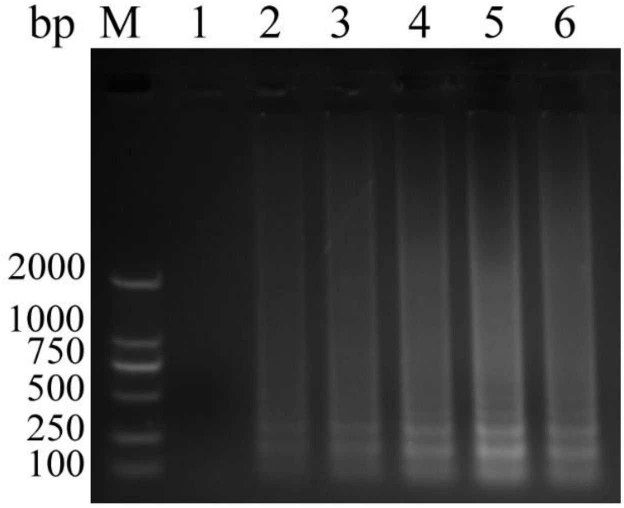

Supplementary Figure S4 Analysis of ALV-J CPA at different concentration of Mg^{2+} by agarose gel electrophoresis (repeated analyze). Lane M, DNA marker; 1, 0 mmol L^{-1}; 2, 1 mmol L^{-1}; 3, 2 mmol L^{-1}; 4, 3 mmol L^{-1}; 5, 4 mmol L^{-1}; 6, 5 mmol L^{-1}. The full-length gel is presented in Supplementary Figure S29.

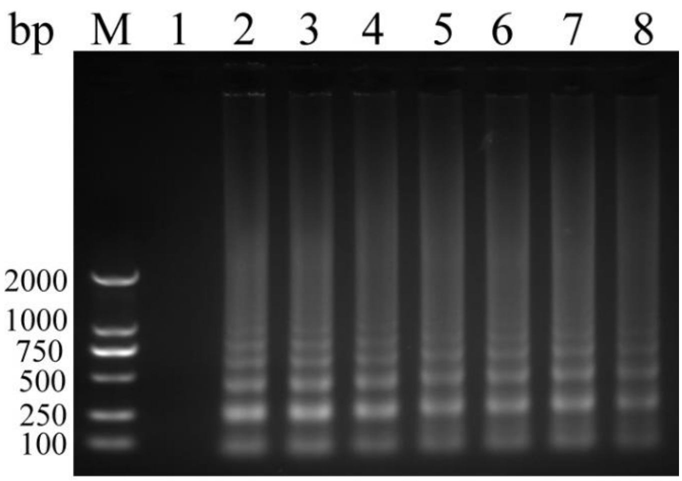

Supplementary Figure S5 Analysis of ALV-J CPA at different concentration of Betaine by agarose gel electrophoresis (repeated analyze). Lane M, DNA marker; 1, 0 mol L^{-1}; 2, 0.2 mol L^{-1}; 3, 0.4 mol L^{-1}; 4, 0.6 mol L^{-1}; 5, 0.8 mol L^{-1}; 6, 1.0 mol L^{-1}; 7, 1.2 mol L^{-1}; 8, 1.4 mol L^{-1}. The full-length gel is presented in Supplementary Figure S30.

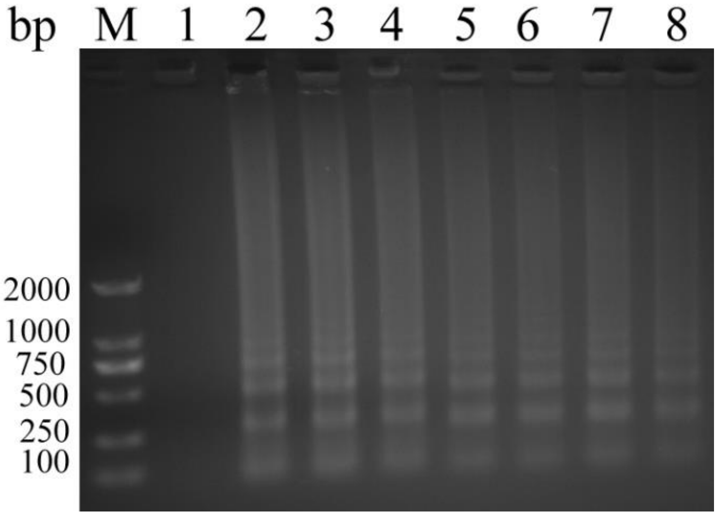

Supplementary Figure S6 Analysis of ALV-J CPA at different concentration of Betaine by agarose gel electrophoresis (repeated analyze). Lane M, DNA marker; 1, 0 mol L^{-1}; 2, 0.2 mol L^{-1}; 3, 0.4 mol L^{-1}; 4, 0.6 mol L^{-1}; 5, 0.8 mol L^{-1}; 6, 1.0 mol L^{-1}; 7, 1.2 mol L^{-1}; 8, 1.4 mol L^{-1}. The full-length gel is presented in Supplementary Figure S31.

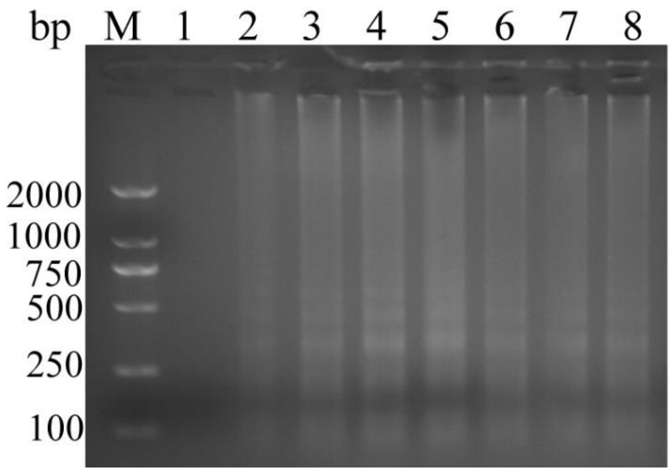

Supplementary Figure S7 Analysis of ALV-J CPA at different concentration of dNTPs by agarose gel electrophoresis (repeated analyze). Lane M, DNA marker; 1, 0 mmol L^{-1}; 2, 0.2 mmol L^{-1}; 3, 0.4 mmol L^{-1}; 4, 0.6 mmol L^{-1}; 5, 0.8 mmol L^{-1}; 6, 1.0 mmol L^{-1}; 7, 1.2 mmol L^{-1}; 8, 1.4 mmol L^{-1}. The full-length gel is presented in Supplementary Figure S32.

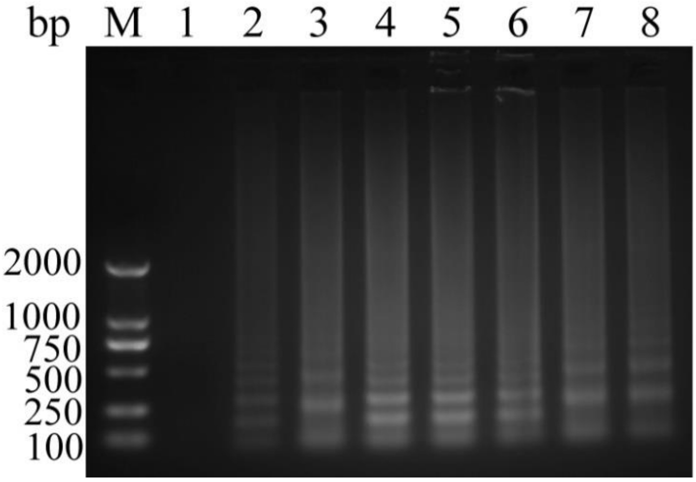

Supplementary Figure S8 Analysis of ALV-J CPA at different concentration of dNTPs by agarose gel electrophoresis (repeated analyze). Lane M, DNA marker; 1, 0 mmol L^{-1}; 2, 0.2 mmol L^{-1}; 3, 0.4 mmol L^{-1}; 4, 0.6 mmol L^{-1}; 5, 0.8 mmol L^{-1}; 6, 1.0 mmol L^{-1}; 7, 1.2 mmol L^{-1}; 8, 1.4 mmol L^{-1}. The full-length gel is presented in Supplementary Figure S33.

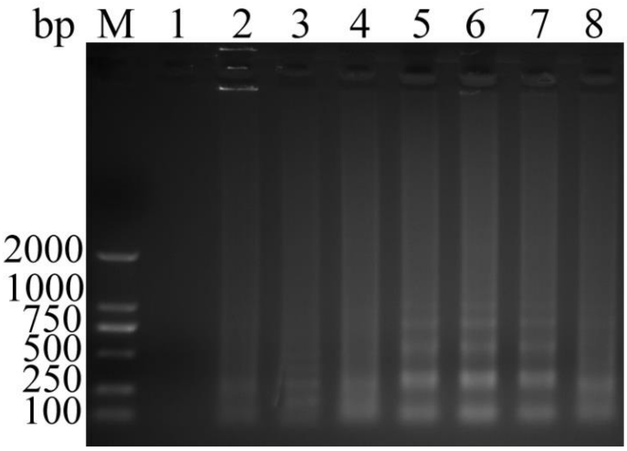

Supplementary Figure S9 Analysis of ALV-J CPA at different units of *Bst* DNA polymerase (8 units μL^{-1}) by agarose gel electrophoresis (repeated analyze). Lane M, DNA markers; 1, 0 units μL^{-1}; 2, 0.064 units μL^{-1}; 3, 0.128 units μL^{-1}; 4, 0.192 units μL^{-1}; 5, 0.256 units μL^{-1}; 6, 0.32 units μL^{-1}; 7, 0.48 units μL^{-1}; 8, 0.64 units μL^{-1}. The full-length gel is presented in Supplementary Figure S34.

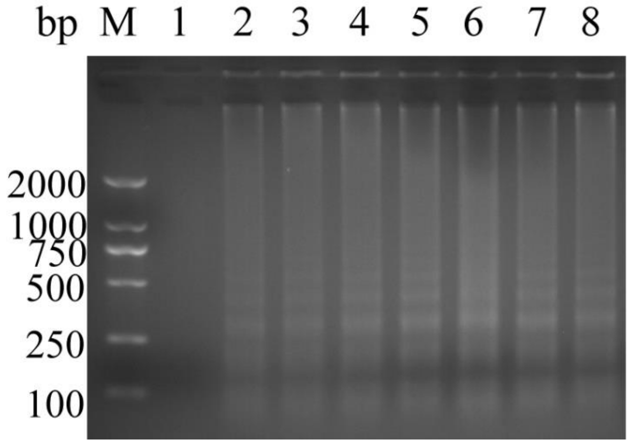

Supplementary Figure S10 Analysis of ALV-J CPA at different units of *Bst* DNA polymerase (8 units μL^{-1}) by agarose gel electrophoresis (repeated analyze). Lane M, DNA markers; 1, 0 units μL^{-1}; 2, 0.064 units μL^{-1}; 3, 0.128 units μL^{-1}; 4, 0.192 units μL^{-1}; 5, 0.256 units μL^{-1}; 6, 0.32 units μL^{-1}; 7, 0.48 units μL^{-1}; 8, 0.64 units μL^{-1}. The full-length gel is presented in Supplementary Figure S35.

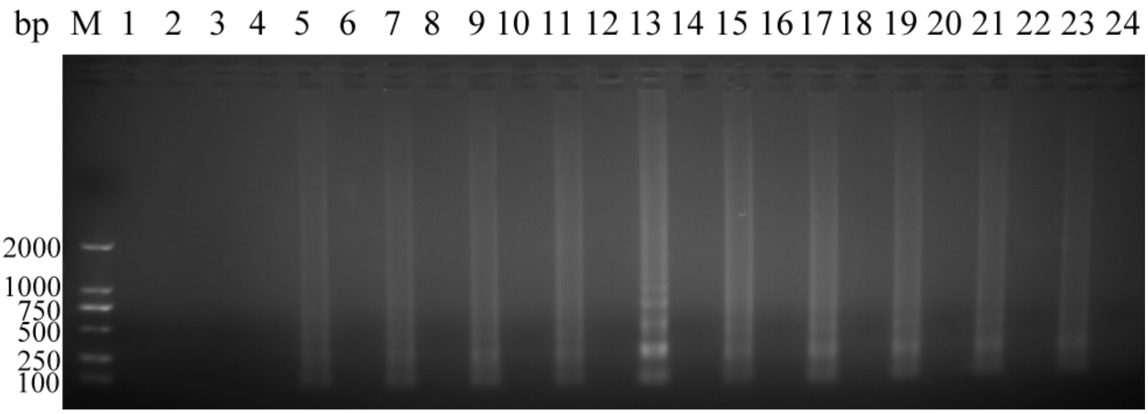

Supplementary Figure S11 Analysis of ALV-J CPA at different temperatures by agarose gel electrophoresis (repeated analyze). Lane M, DNA marker; 1, 54°C; 3, 55°C; 5, 56°C; 7, 57°C; 9, 58°C; 11, 59°C; 13, 60°C; 15, 61°C; 17, 62°C; 19, 63°C; 21, 64°C; 23, 65°C; 2, 4, 6, 8, 10, 12, 14, 16, 18, 20, 22 and 24 were negative controls for the corresponding temperatures. The full-length gel is presented in Supplementary Figure S36.

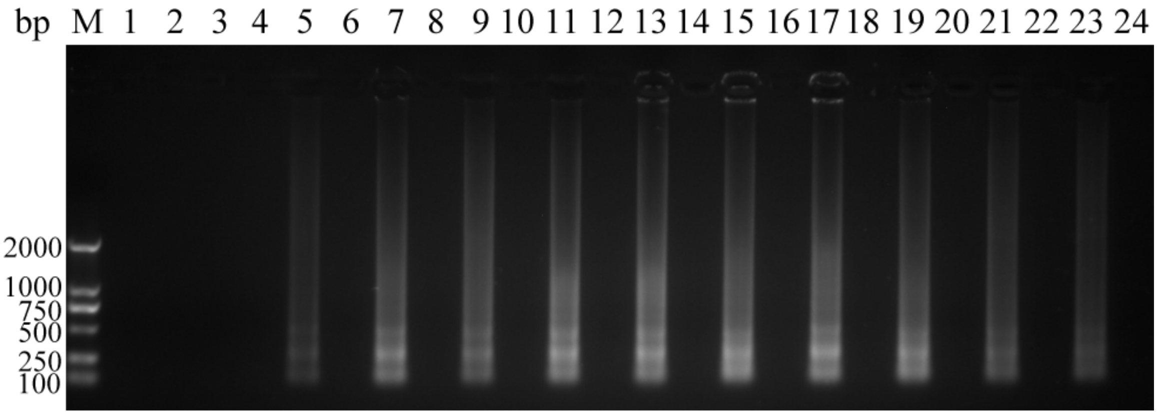

Supplementary Figure S12 Analysis of ALV-J CPA at different temperatures by agarose gel electrophoresis (repeated analyze). Lane M, DNA marker; 1, 54°C; 3, 55°C; 5, 56°C; 7, 57°C; 9, 58°C; 11, 59°C; 13, 60°C; 15, 61°C; 17, 62°C; 19, 63°C; 21, 64°C; 23, 65°C; 2, 4, 6, 8, 10, 12, 14, 16, 18, 20, 22 and 24 were negative controls for the corresponding temperatures. The full-length gel is presented in Supplementary Figure S37.

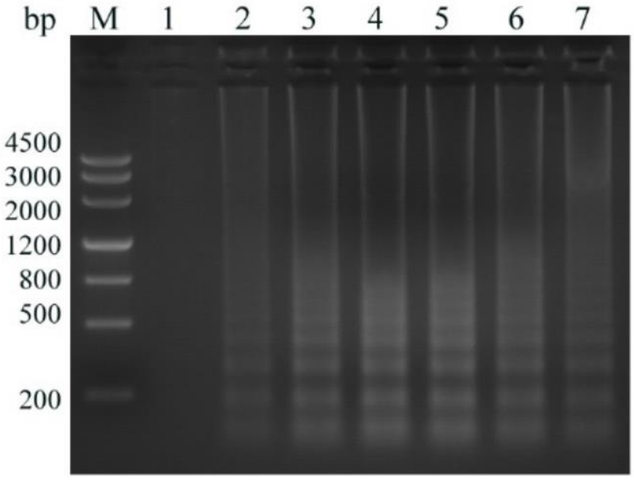

Supplementary Figure S13 Analysis of ALV-J CPA with different reaction times (30min -75min) agarose gel electrophoresis. Lane M, DNA marker; 1, Negative control amplification for 75 min; 2, 30 min; 3, 45 min; 4, 60 min; 5, 65 min; 6, 70 min; 7, 75 min. The full-length gel is presented in Supplementary Figure S38.

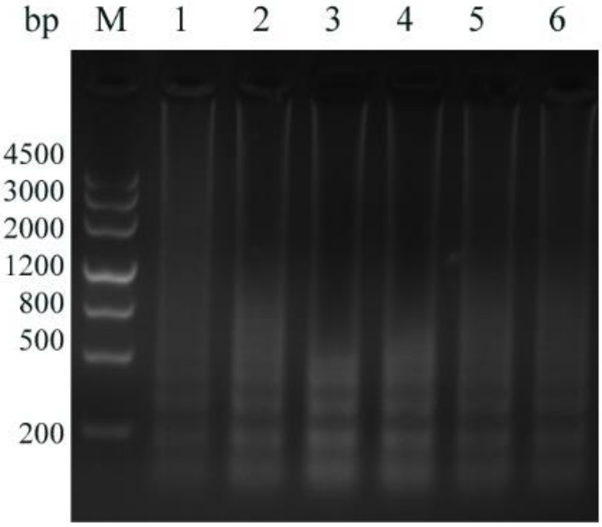

Supplementary Figure S14 Analysis of ALV-J CPA with different reaction times (30min -75min) by agarose gel electrophoresis (repeated analyze). Lane M, DNA marker; 1, 30 min; 2, 45 min; 3, 60 min; 4, 65 min; 5, 70 min; 6, 75 min. The full-length gel is presented in Supplementary Figure S39.

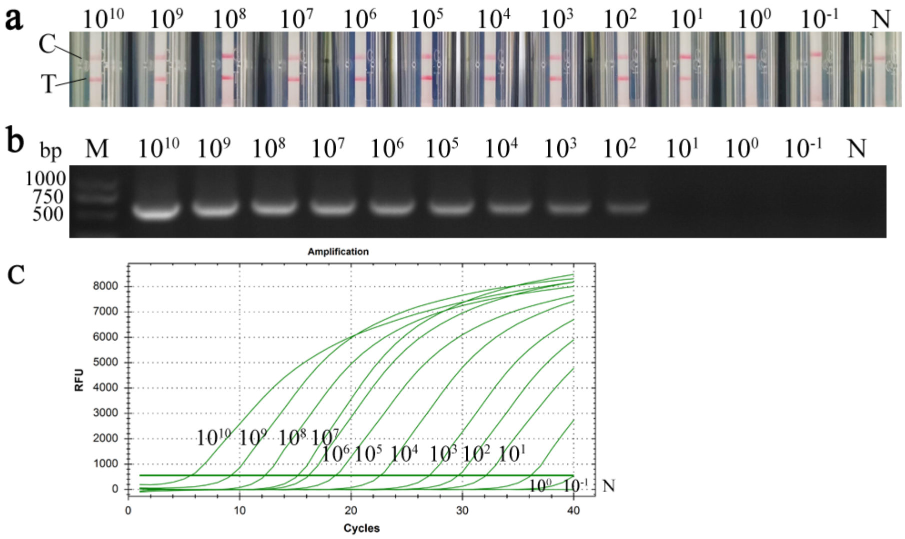

Supplementary Figure S15 Comparison of sensitivity between the CPA, PCR and RT-PCR of ALV-J detect methods based on recombinant plasmid standards at different copy numbers (1.86×10^{-1} ~ 1.86×10^{10} copies μL^{-1}) (repeated analyze). (**a**) Sensitivity of CPA based on a nucleic acid detection device; (**b**) Sensitivity of conventional PCR; The full-length gel is presented in Supplementary Figure S40. (**c**) Sensitivity of RT-PCR for ALV-J. C, control-line; T, test-line; N, negative control.

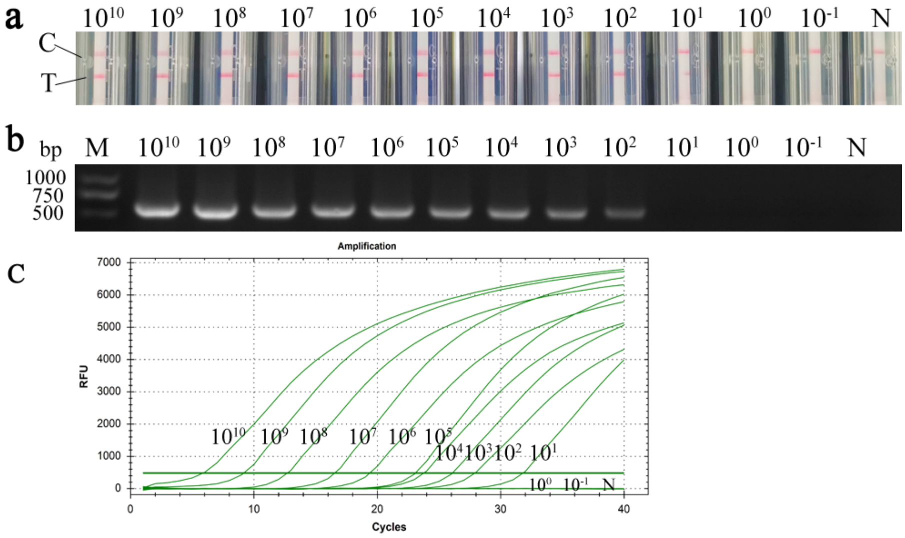

Supplementary Figure S16 Comparison of sensitivity between the CPA, PCR and RT-PCR of ALV-J detect methods based on recombinant plasmid standards at different copy numbers (1.86×10^{-1} ~ 1.86×10^{10} copies μL^{-1}) (repeated analyze). (**a**) Sensitivity of CPA based on a nucleic acid detection device; (**b**) Sensitivity of conventional PCR; The full-length gel is presented in Supplementary Figure S41. (**c**) Sensitivity of RT-PCR for ALV-J. C, control-line; T, test-line; N, negative control.

The full-length gels used in the main article which were cropped:

Supplementary Figure S17

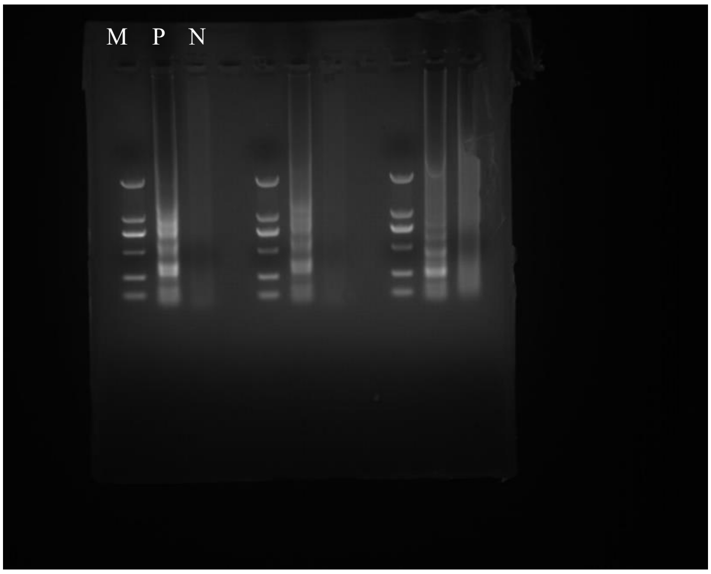

Supplementary Figure S17 The full-length gel of Fig. 1a in the main article. Agarose gel electrophoresis of test samples; Lane M, DNA markers; P, ALV-J; N, negative control.

Supplementary Figure S18

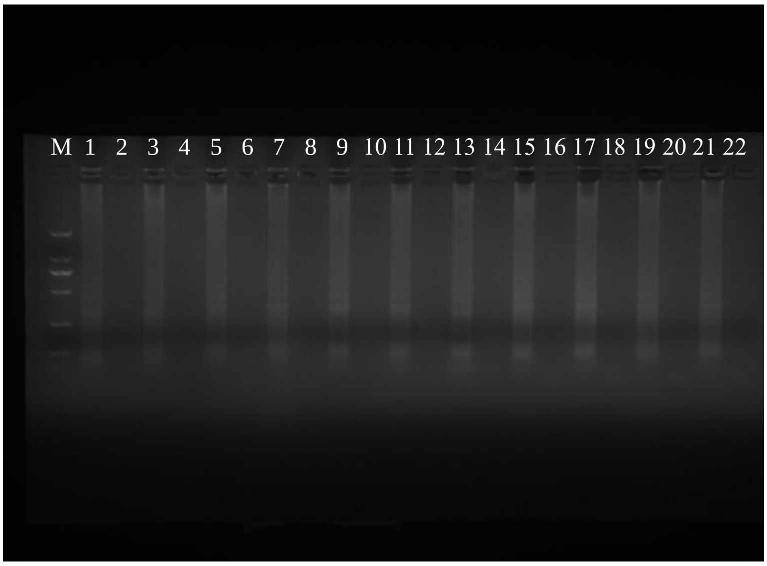

Supplementary Figure S18 The full-length gel of Fig. 2 in the main article. Analysis of ALV-J CPA at different concentration of primers (Table 1) by agarose gel electrophoresis. Lane M, DNA marker; 1, 3, 5, 7, 9, 11, 13, 15, 17, 19, 21, were primers groups 1-11 (Table 1) respectively; 2, 4, 6, 8, 10, 12, 14, 16, 18, 20 and 22 were negative controls for the corresponding primers groups.

Supplementary Figure S19

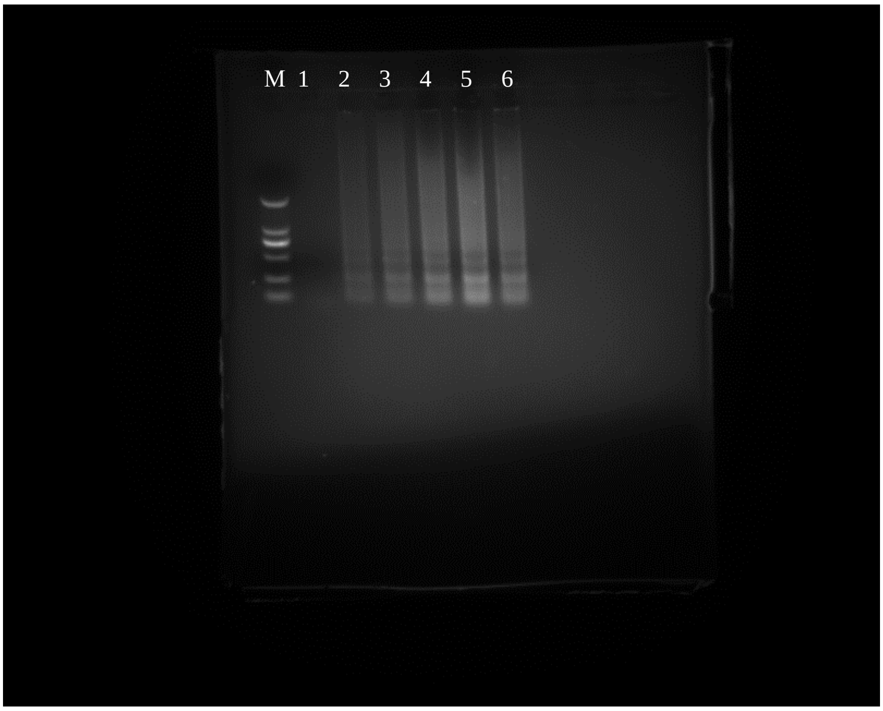

Supplementary Figure S19 The full-length gel of Fig. 3 in the main article. Analysis of ALV-J CPA at different concentration of Mg^{2+} by agarose gel electrophoresis. Lane M, DNA marker; 1, 0 mmol L^{-1}; 2, 1 mmol L^{-1}; 3, 2 mmol L^{-1}; 4, 3 mmol L^{-1}; 5, 4 mmol L^{-1}; 6, 5 mmol L^{-1}.

Supplementary Figure S20

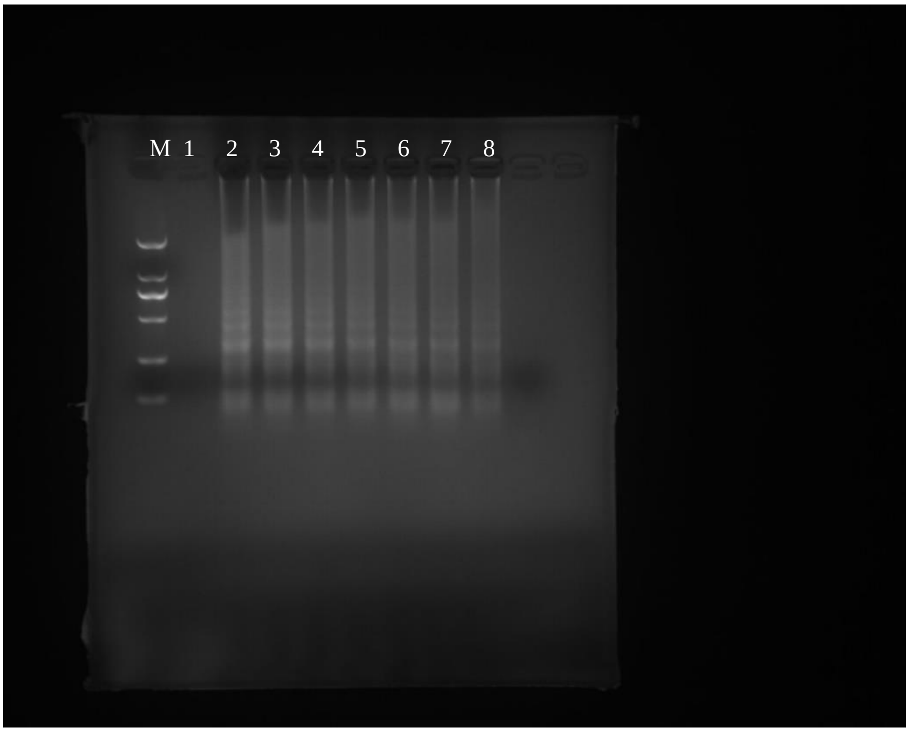

Supplementary Figure S20 The full-length gel of Fig. 4 in the main article. Analysis of ALV-J CPA at different concentration of Betaine by agarose gel electrophoresis. Lane M, DNA marker; 1, 0 mol L^{-1}; 2, 0.2 mol L^{-1}; 3, 0.4 mol L^{-1}; 4, 0.6 mol L^{-1}; 5, 0.8 mol L^{-1}; 6, 1.0 mol L^{-1}; 7, 1.2 mol L^{-1}; 8, 1.4 mol L^{-1}.

Supplementary Figure S21

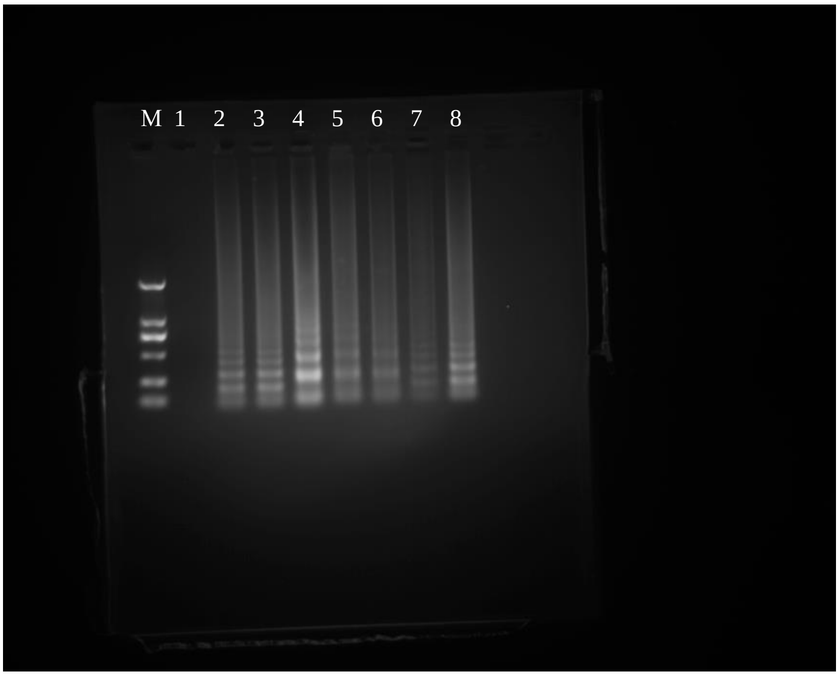

Supplementary Figure S21 The full-length gel of Fig. 5 in the main article. Analysis of ALV-J CPA at different concentration of dNTPs by agarose gel electrophoresis. Lane M, DNA marker; 1, 0 mmol L^{-1}; 2, 0.2 mmol L^{-1}; 3, 0.4 mmol L^{-1}; 4, 0.6 mmol L^{-1}; 5, 0.8 mmol L^{-1}; 6, 1.0 mmol L^{-1}; 7, 1.2 mmol L^{-1}; 8, 1.4 mmol L^{-1}.

Supplementary Figure S22

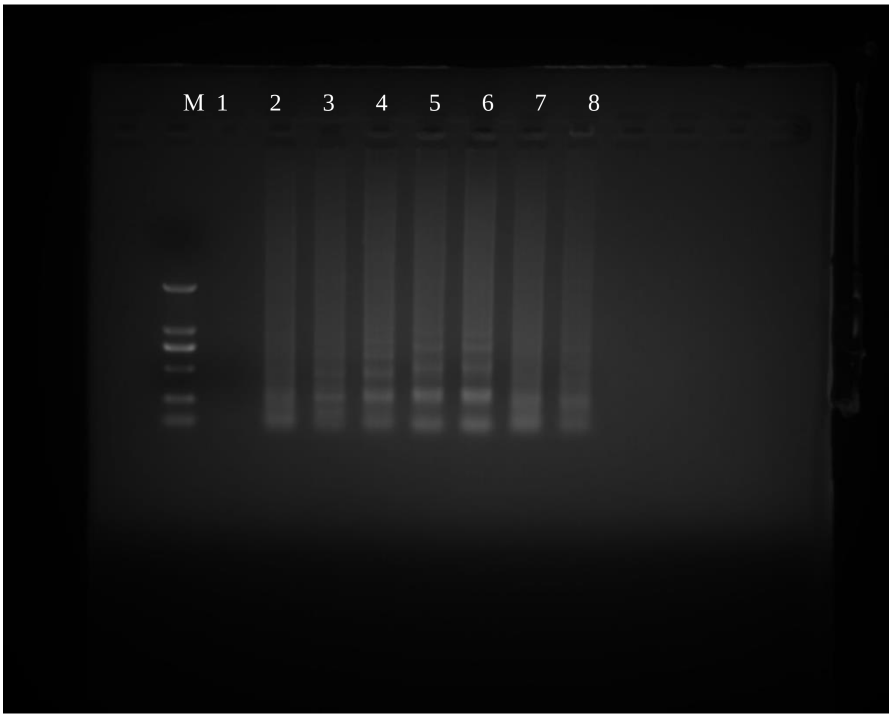

Supplementary Figure S22 The full-length gel of Fig. 6 in the main article. Analysis of ALV-J CPA at different units of *Bst* DNA polymerase (8 units μL^{-1}) by agarose gel electrophoresis. Lane M, DNA markers; 1, 0 units μL^{-1}; 2, 0.064 units μL^{-1}; 3, 0.128 units μL^{-1}; 4, 0.192 units μL^{-1}; 5, 0.256 units μL^{-1}; 6, 0.32 units μL^{-1}; 7, 0.48 units μL^{-1}; 8, 0.64 units μL^{-1}.

Supplementary Figure S23

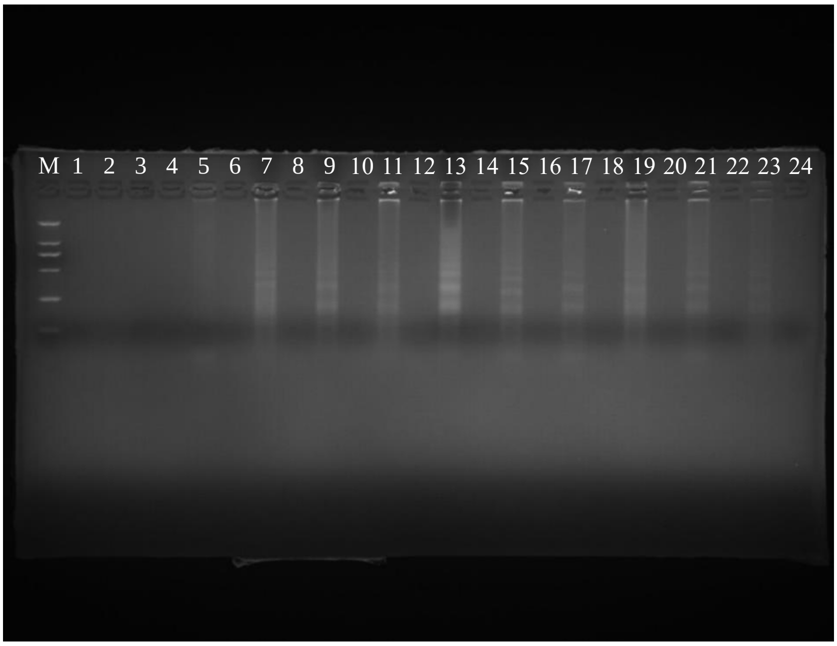

Supplementary Figure S23 The full-length gel of Fig. 7 in the main article. Analysis of ALV-J CPA at different temperatures by agarose gel electrophoresis. Lane M, DNA marker; 1, 54°C; 3, 55°C; 5, 56°C; 7, 57°C; 9, 58°C; 11, 59°C; 13, 60°C; 15, 61°C; 17, 62°C; 19, 63°C; 21, 64°C; 23, 65°C; 2, 4, 6, 8, 10, 12, 14, 16, 18, 20, 22 and 24 were negative controls for the corresponding temperatures.

Supplementary Figure S24

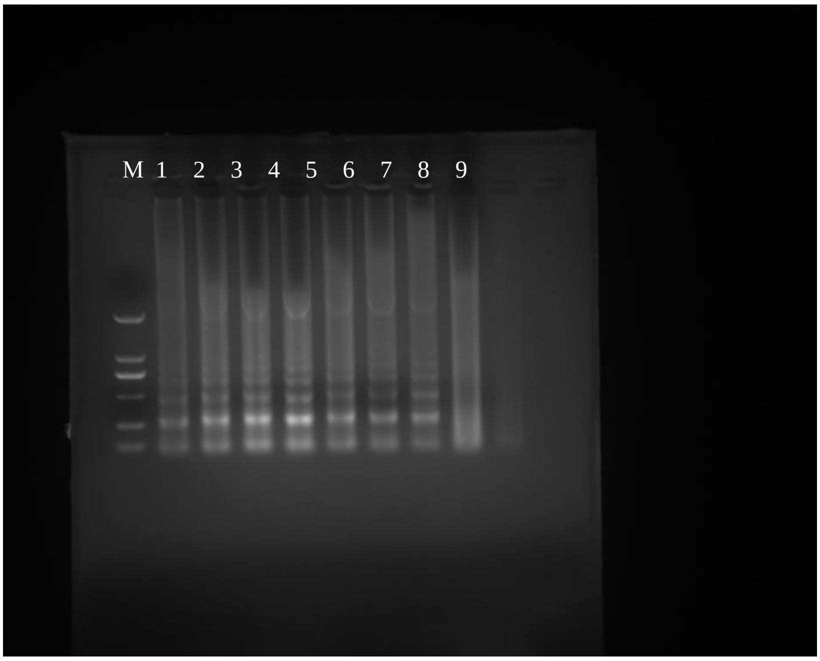

Supplementary Figure S24 The full-length gel of Fig. 8 in the main article. Analysis of ALV-J CPA with different reaction times by agarose gel electrophoresis. Lane M, DNA marker; 1, 15 min; 2, 30 min; 3, 45 min; 4, 60 min; 5, 75 min; 6, 90 min; 7, 105 min; 8, 120 min; 9, Negative control amplification for 120 min.

Supplementary Figure S25

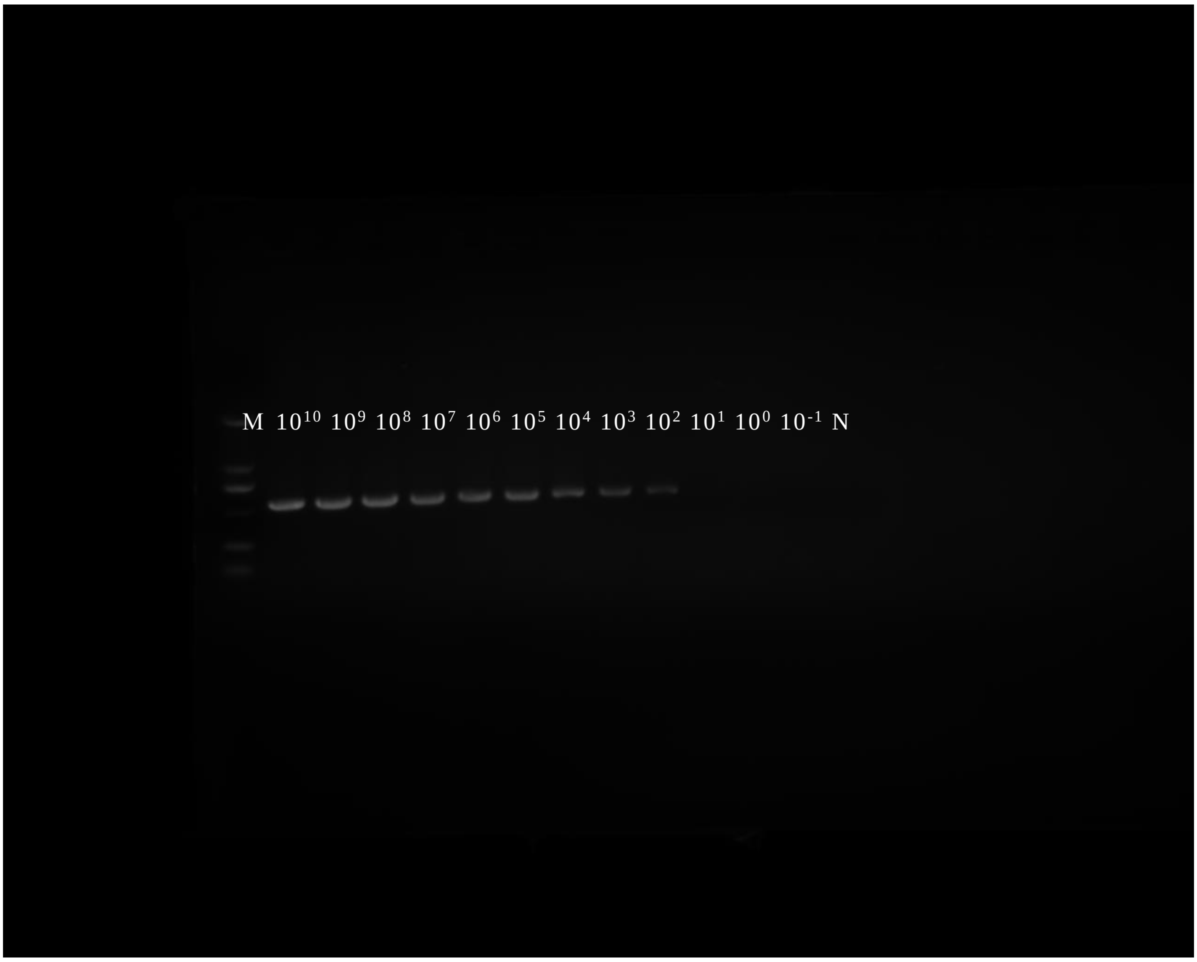

Supplementary Figure S25 The full-length gel of Fig. 10b in the main article. Sensitivity of conventional PCR. Lane M, DNA markers; N, negative control.

The full-length gels used in the <u>Supplementary Figures</u> which were cropped:

Supplementary Figure S26

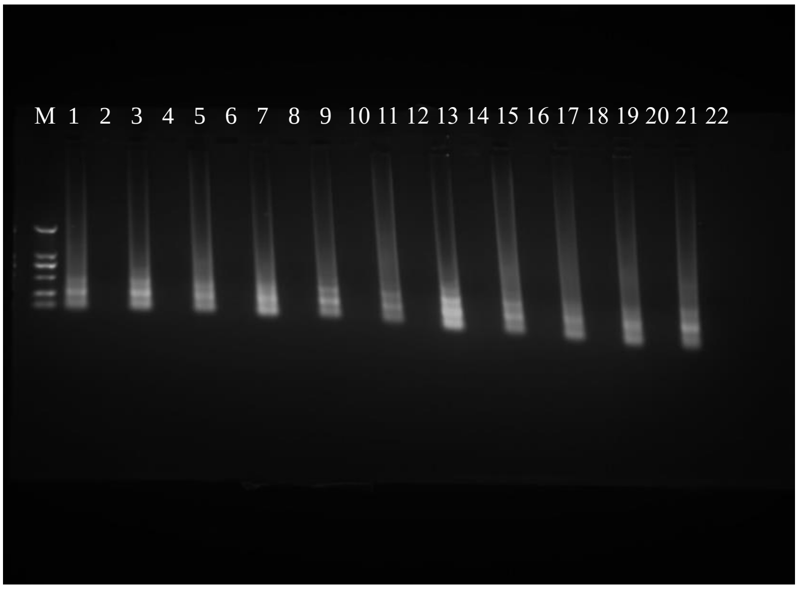

Supplementary Figure S26 The full-length gel of Supplementary Fig. S1 in the supplementary information. Analysis of ALV-J CPA at different concentration of primers (Table 1) by agarose gel electrophoresis (repeated analyze). Lane M, DNA marker; 1, 3, 5, 7, 9, 11, 13, 15, 17, 19, 21, were primers groups 1-11 (Table 1) respectively; 2, 4, 6, 8, 10, 12, 14, 16, 18, 20 and 22 were negative controls for the corresponding primers groups.

Supplementary Figure S27

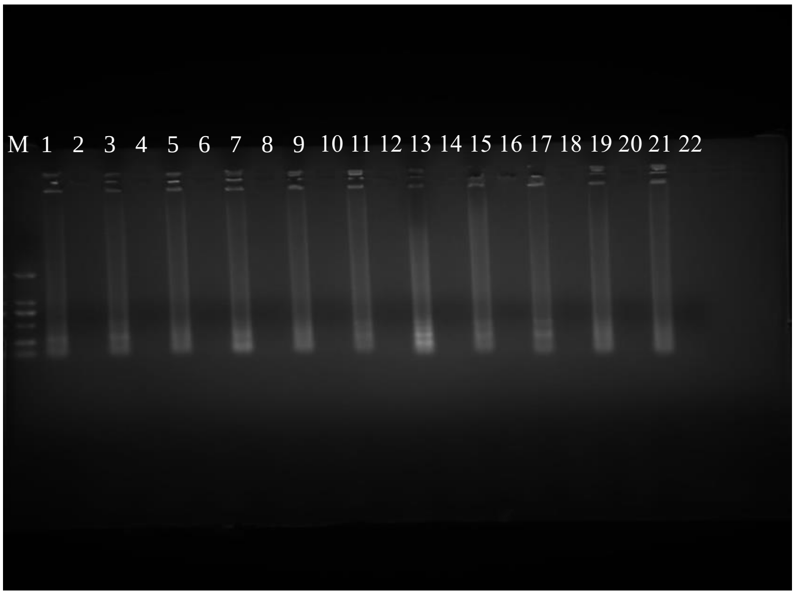

Supplementary Figure S27 The full-length gel of Supplementary Fig. S2 in the supplementary information. Analysis of ALV-J CPA at different concentration of primers (Table 1) by agarose gel electrophoresis (repeated analyze). Lane M, DNA marker; 1, 3, 5, 7, 9, 11, 13, 15, 17, 19, 21, were primers groups 1-11 (Table 1) respectively; 2, 4, 6, 8, 10, 12, 14, 16, 18, 20 and 22 were negative controls for the corresponding primers groups.

Supplementary Figure S28

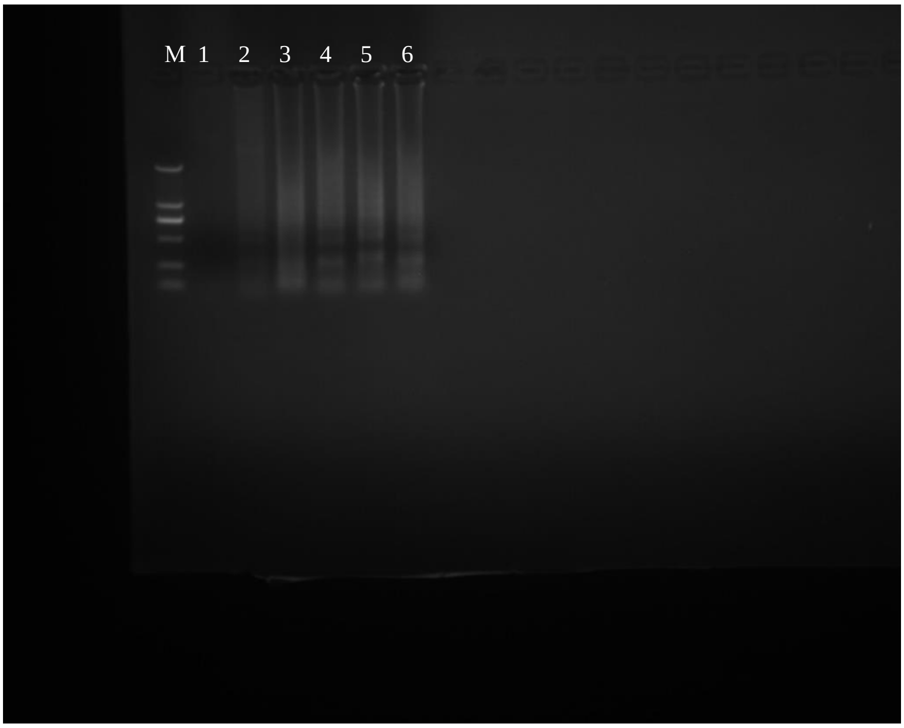

Supplementary Figure S28 The full-length gel of Supplementary Fig. S3 in the supplementary information. Analysis of ALV-J CPA at different concentration of Mg^{2+} by agarose gel electrophoresis (repeated analyze). Lane M, DNA marker; 1, 0 mmol L^{-1}; 2, 1 mmol L^{-1}; 3, 2 mmol L^{-1}; 4, 3 mmol L^{-1}; 5, 4 mmol L^{-1}; 6, 5 mmol L^{-1}.

Supplementary Figure S29

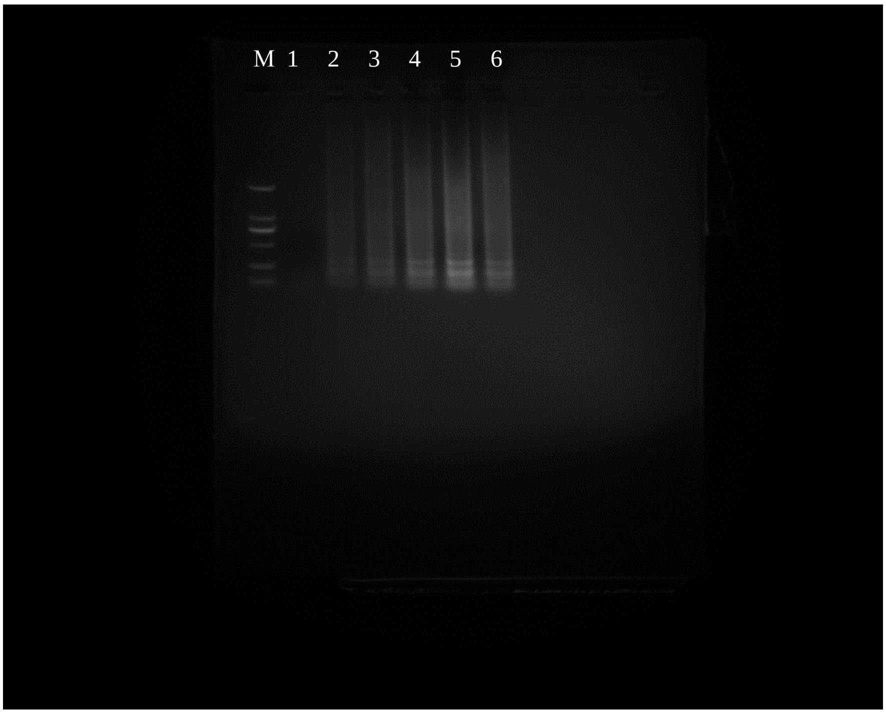

Supplementary Figure S29 The full-length gel of Supplementary Fig. S4 in the supplementary information. Analysis of ALV-J CPA at different concentration of Mg^{2+} by agarose gel electrophoresis (repeated analyze). Lane M, DNA marker; 1, 0 mmol L^{-1}; 2, 1 mmol L^{-1}; 3, 2 mmol L^{-1}; 4, 3 mmol L^{-1}; 5, 4 mmol L^{-1}; 6, 5 mmol L^{-1}.

Supplementary Figure S30

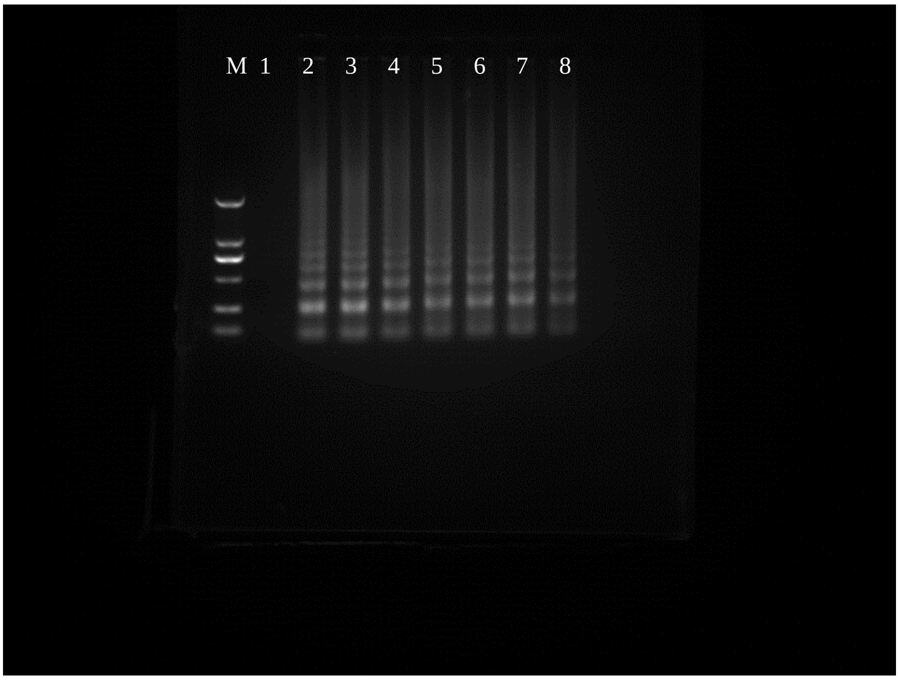

Supplementary Figure S30 The full-length gel of Supplementary Fig. S5 in the supplementary information. Analysis of ALV-J CPA at different concentration of Betaine by agarose gel electrophoresis (repeated analyze). Lane M, DNA marker; 1, 0 mol L^{-1}; 2, 0.2 mol L^{-1}; 3, 0.4 mol L^{-1}; 4, 0.6 mol L^{-1}; 5, 0.8 mol L^{-1}; 6, 1.0 mol L^{-1}; 7, 1.2 mol L^{-1}; 8, 1.4 mol L^{-1}.

Supplementary Figure S31

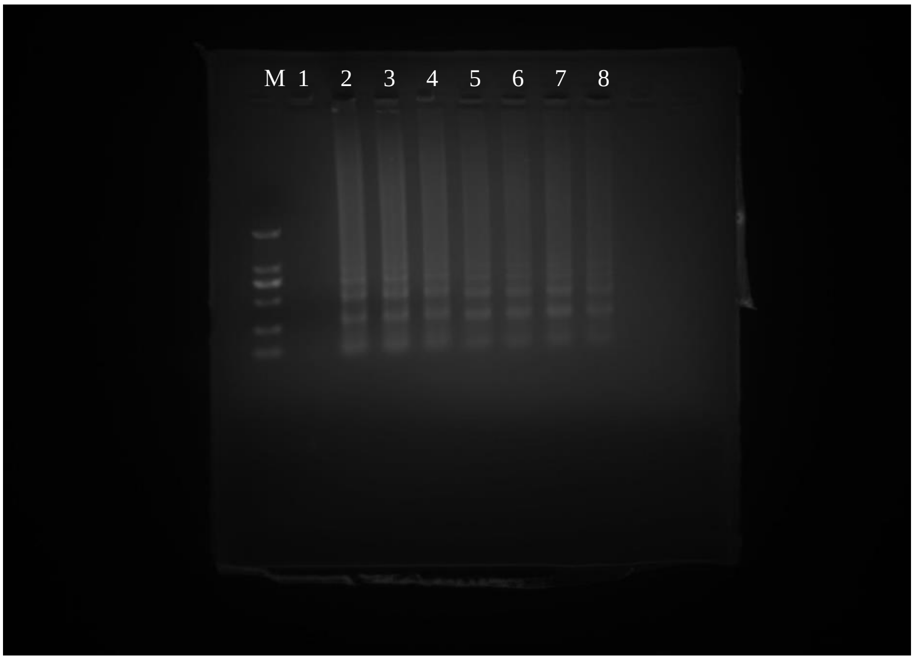

Supplementary Figure S31 The full-length gel of Supplementary Fig. S6 in the supplementary information. Analysis of ALV-J CPA at different concentration of Betaine by agarose gel electrophoresis (repeated analyze). Lane M, DNA marker; 1, 0 mol L^{-1}; 2, 0.2 mol L^{-1}; 3, 0.4 mol L^{-1}; 4, 0.6 mol L^{-1}; 5, 0.8 mol L^{-1}; 6, 1.0 mol L^{-1}; 7, 1.2 mol L^{-1}; 8, 1.4 mol L^{-1}.

Supplementary Figure S32

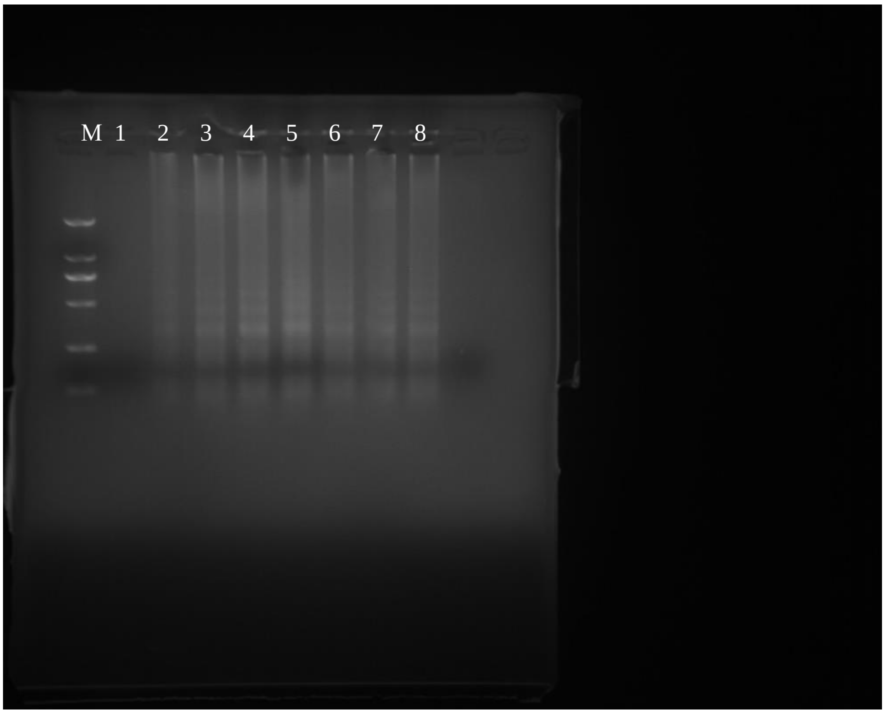

Supplementary Figure S32 The full-length gel of Supplementary Fig. S7 in the supplementary information. Analysis of ALV-J CPA at different concentration of dNTPs by agarose gel electrophoresis (repeated analyze). Lane M, DNA marker; 1, 0 mmol L^{-1}; 2, 0.2 mmol L^{-1}; 3, 0.4 mmol L^{-1}; 4, 0.6 mmol L^{-1}; 5, 0.8 mmol L^{-1}; 6, 1.0 mmol L^{-1}; 7, 1.2 mmol L^{-1}; 8, 1.4 mmol L^{-1}.

Supplementary Figure S33

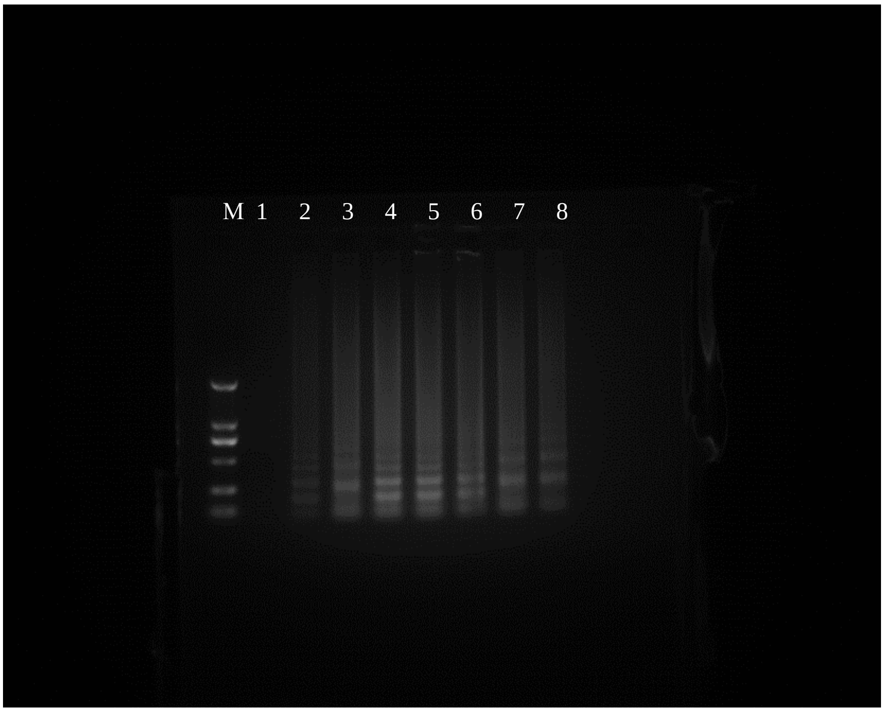

Supplementary Figure S33 The full-length gel of Supplementary Fig. S8 in the supplementary information. Analysis of ALV-J CPA at different concentration of dNTPs by agarose gel electrophoresis (repeated analyze). Lane M, DNA marker; 1, 0 mmol L^{-1}; 2, 0.2 mmol L^{-1}; 3, 0.4 mmol L^{-1}; 4, 0.6 mmol L^{-1}; 5, 0.8 mmol L^{-1}; 6, 1.0 mmol L^{-1}; 7, 1.2 mmol L^{-1}; 8, 1.4 mmol L^{-1}.

Supplementary Figure S34

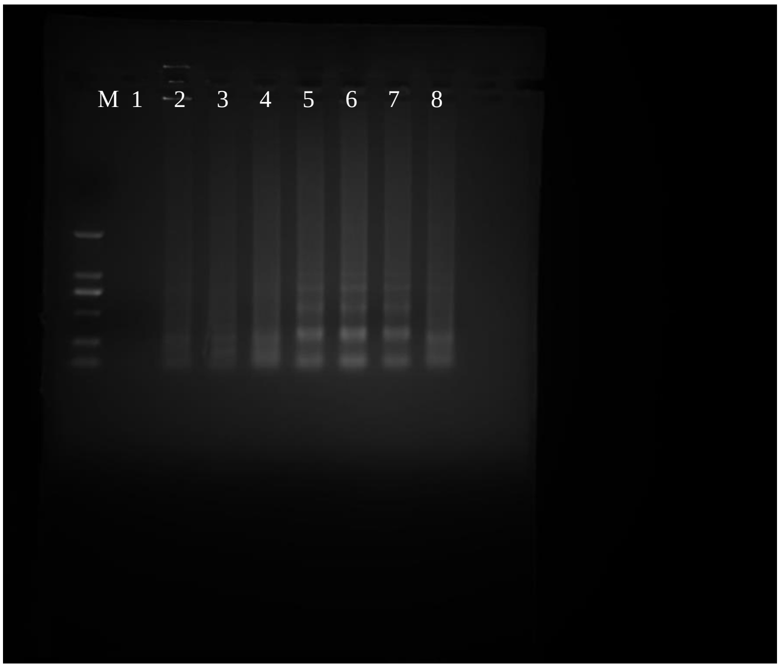

Supplementary Figure S34 The full-length gel of Supplementary Fig. S9 in the supplementary information. Analysis of ALV-J CPA at different units of *Bst* DNA polymerase (8 units μL^{-1}) by agarose gel electrophoresis (repeated analyze). Lane M, DNA markers; 1, 0 units μL^{-1}; 2, 0.064 units μL^{-1}; 3, 0.128 units μL^{-1}; 4, 0.192 units μL^{-1}; 5, 0.256 units μL^{-1}; 6, 0.32 units μL^{-1}; 7, 0.48 units μL^{-1}; 8, 0.64 units μL^{-1}.

Supplementary Figure S35

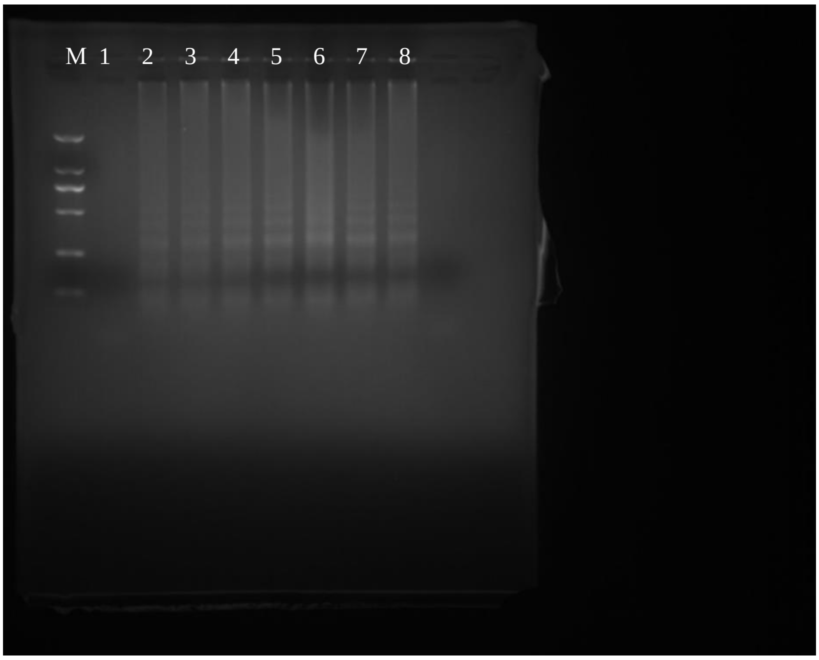

Supplementary Figure S35 The full-length gel of Supplementary Fig. S10 in the supplementary information. Analysis of ALV-J CPA at different units of *Bst* DNA polymerase (8 units μL^{-1}) by agarose gel electrophoresis (repeated analyze). Lane M, DNA markers; 1, 0 units μL^{-1}; 2, 0.064 units μL^{-1}; 3, 0.128 units μL^{-1}; 4, 0.192 units μL^{-1}; 5, 0.256 units μL^{-1}; 6, 0.32 units μL^{-1}; 7, 0.48 units μL^{-1}; 8, 0.64 units μL^{-1}.

Supplementary Figure S36

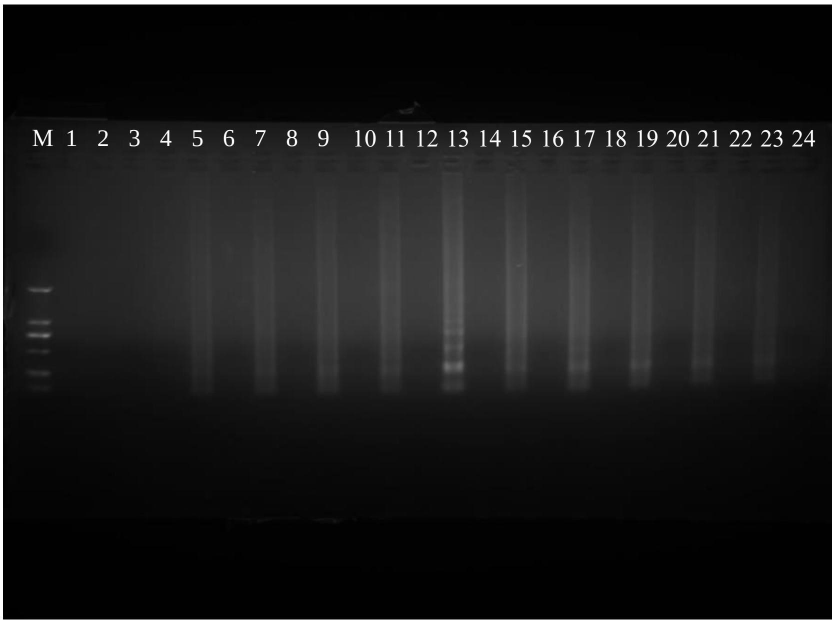

Supplementary Figure S36 The full-length gel of Supplementary Fig. S11 in the supplementary information. Analysis of ALV-J CPA at different temperatures by agarose gel electrophoresis (repeated analyze). Lane M, DNA marker; 1, 54°C; 3, 55°C; 5, 56°C; 7, 57°C; 9, 58°C; 11, 59°C; 13, 60°C; 15, 61°C; 17, 62°C; 19, 63°C; 21, 64°C; 23, 65°C; 2, 4, 6, 8, 10, 12, 14, 16, 18, 20, 22 and 24 were negative controls for the corresponding temperatures.

Supplementary Figure S37

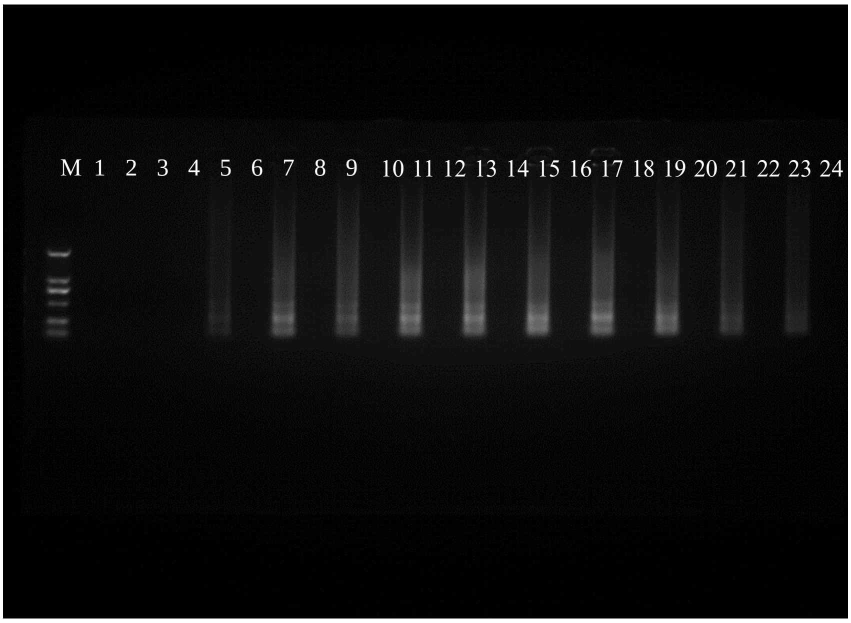

Supplementary Figure S37 The full-length gel of Supplementary Fig. S12 in the supplementary information. Analysis of ALV-J CPA at different temperatures by agarose gel electrophoresis (repeated analyze). Lane M, DNA marker; 1, 54°C; 3, 55°C; 5, 56°C; 7, 57°C; 9, 58°C; 11, 59°C; 13, 60°C; 15, 61°C; 17, 62°C; 19, 63°C; 21, 64°C; 23, 65°C; 2, 4, 6, 8, 10, 12, 14, 16, 18, 20, 22 and 24 were negative controls for the corresponding temperatures.

Supplementary Figure S38

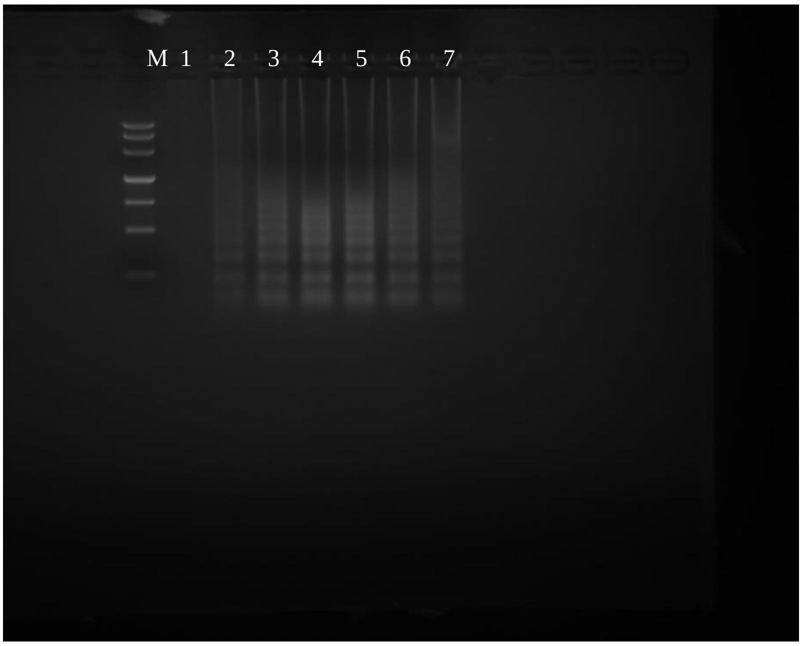

Supplementary Figure S38 The full-length gel of Supplementary Fig. S13 in the supplementary information. Analysis of ALV-J CPA with different reaction times (30min -75min) agarose gel electrophoresis. Lane M, DNA marker; 1, Negative control amplification for 75 min; 2, 30 min; 3, 45 min; 4, 60 min; 5, 65 min; 6, 70 min; 7, 75 min.

Supplementary Figure S39

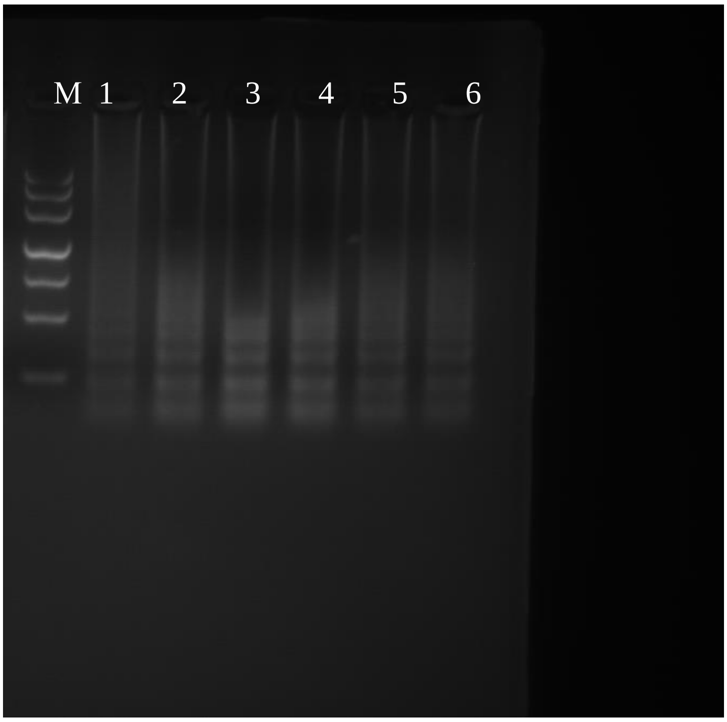

Supplementary Figure S39 The full-length gel of Supplementary Fig. S14 in the supplementary information. Analysis of ALV-J CPA with different reaction times (30min -75min) by agarose gel electrophoresis (repeated analyze). Lane M, DNA marker; 1, 30 min; 2, 45 min; 3, 60 min; 4, 65 min; 5, 70 min; 6, 75 min.

Supplementary Figure S40

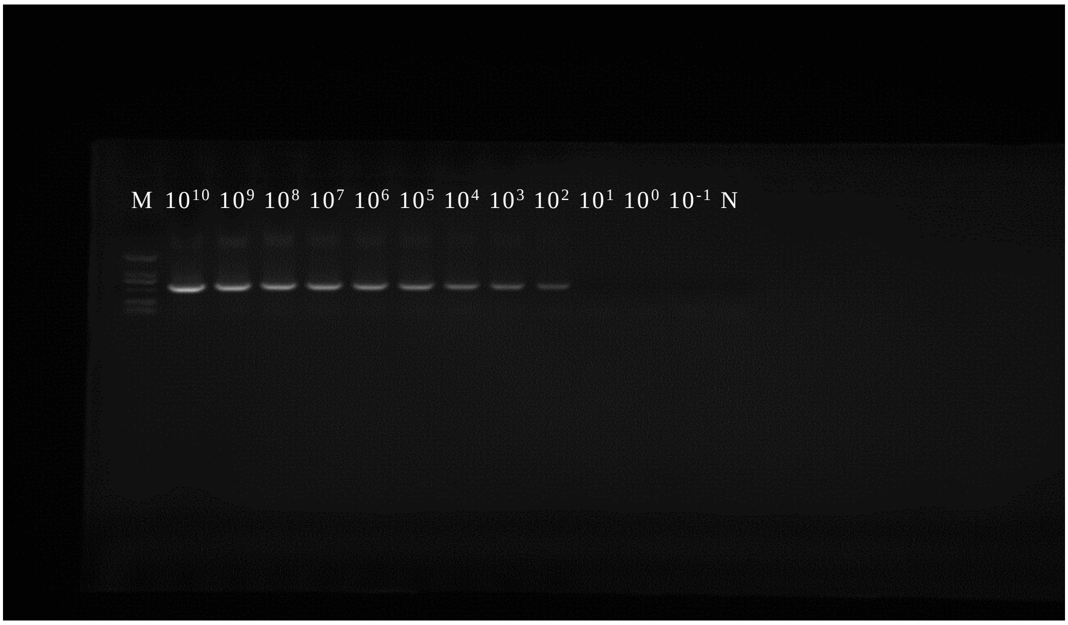

Supplementary Figure S40 The full-length gel of Supplementary Fig. S15 in the supplementary information. Sensitivity of conventional PCR. Lane M, DNA markers; N, negative control.

Supplementary Figure S41

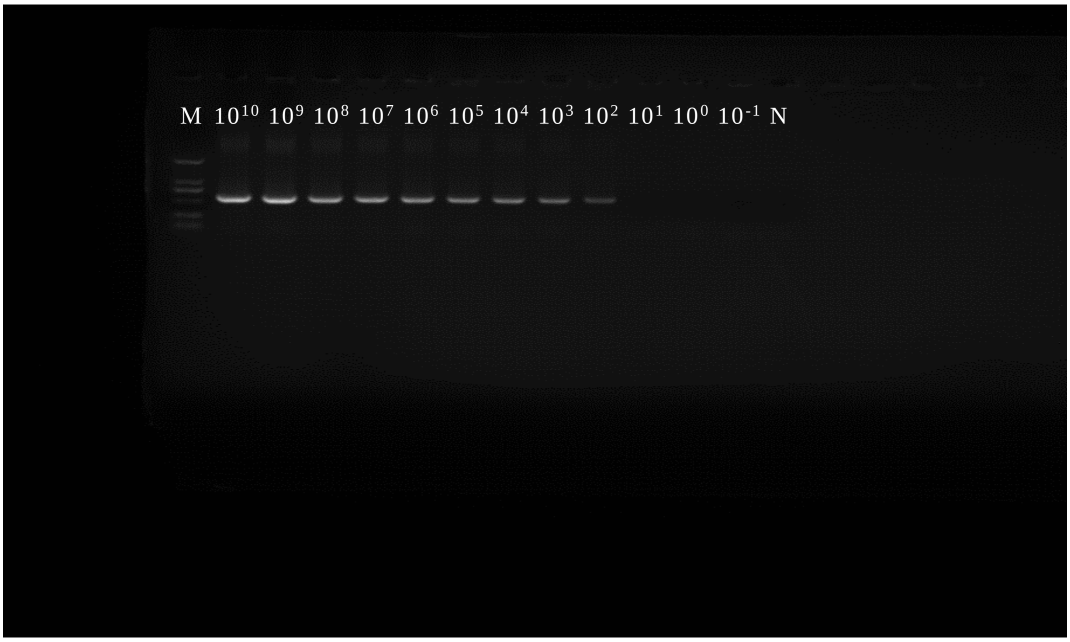

Supplementary Figure S41 The full-length gel of Supplementary Fig. S16 in the supplementary information. Sensitivity of conventional PCR; Lane M, DNA markers; N, negative control.

Supplementary Figure S42

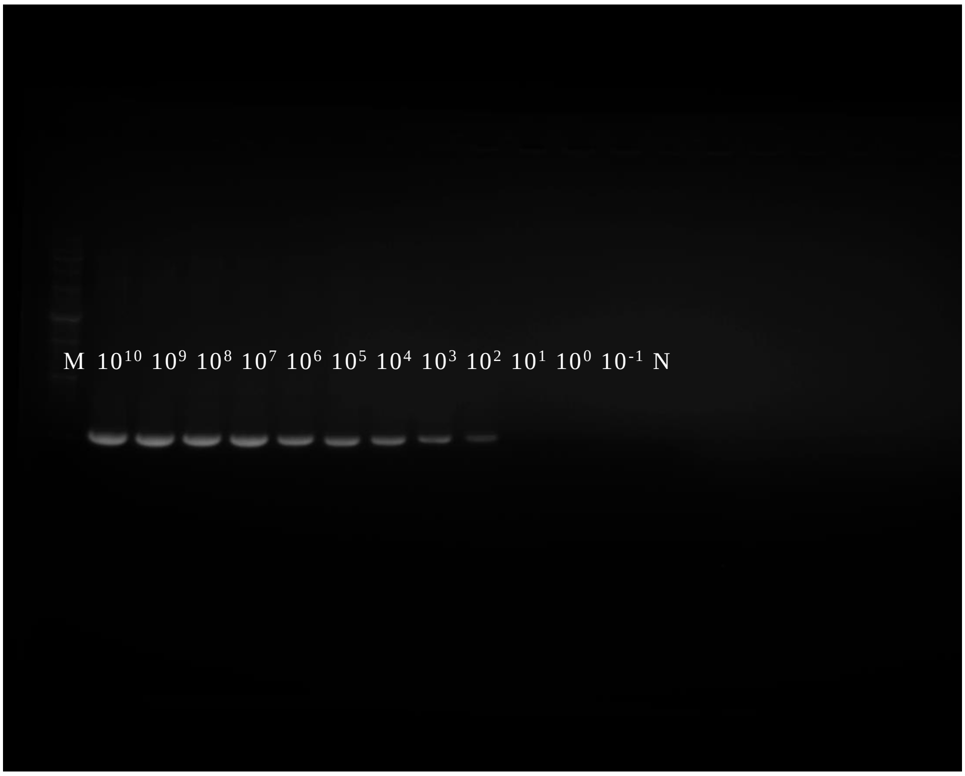

Supplementary Figure S42 Sensitivity of conventional PCR to detect ALV-J (repeated analyze); Lane M, DNA markers; N, negative control.

Supplementary Figure S43

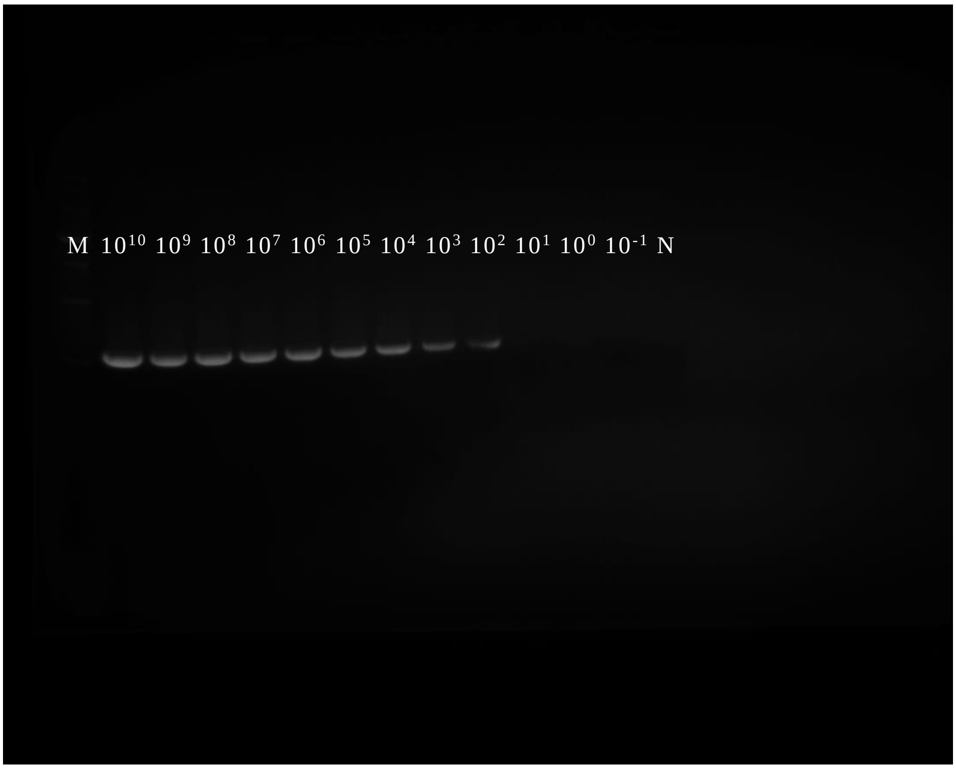

Supplementary Figure S43 Sensitivity of conventional PCR to detect ALV-J (repeated analyze); Lane M, DNA markers; N, negative control.

Supplementary Figure S44

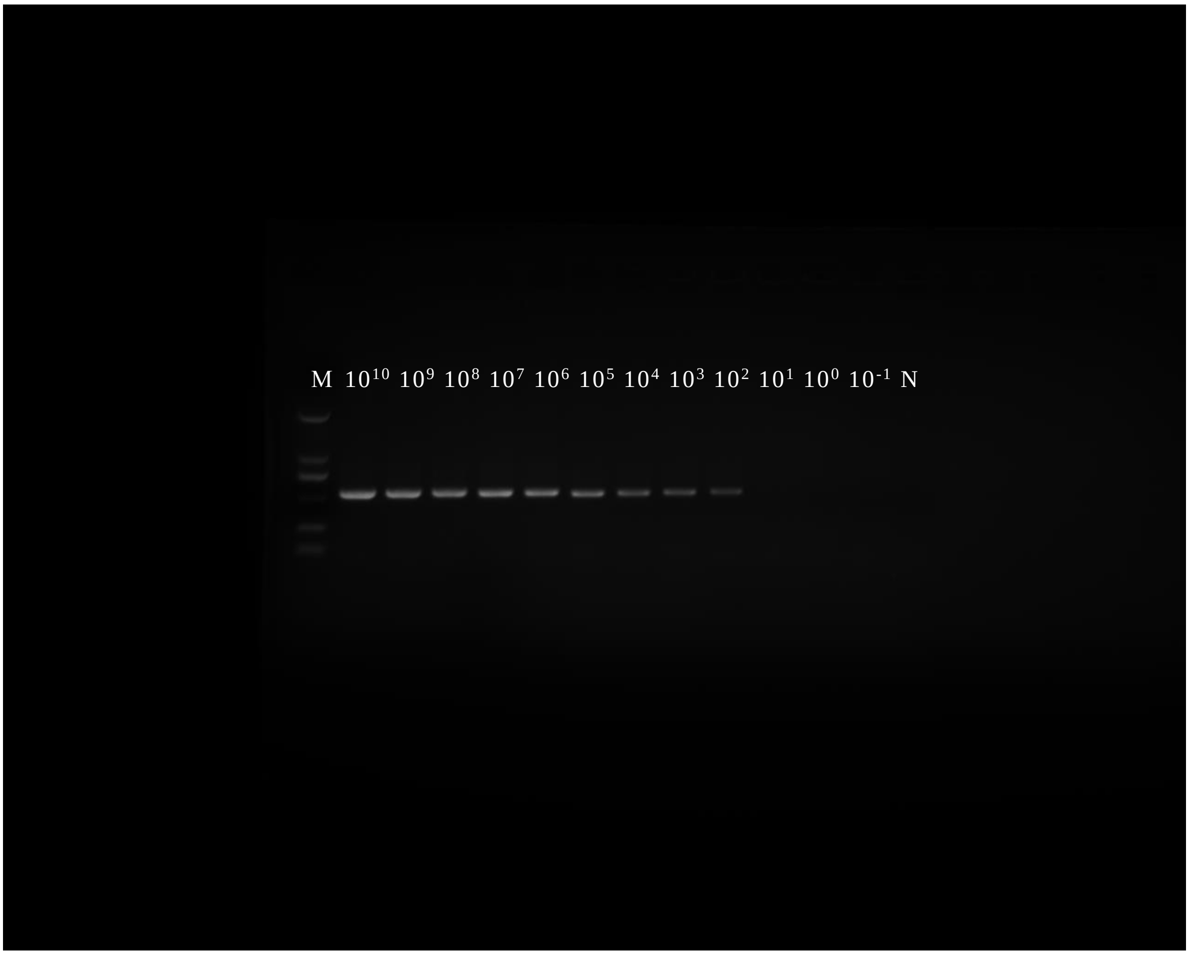

Supplementary Figure S44 Sensitivity of conventional PCR to detect ALV-J (repeated analyze); Lane M, DNA markers; N, negative control.

Supplementary Tables:

Supplementary Table S1 Detection results for cell cultures inoculated with clinical plasma with S/P >0.2

Sample No.	Three methods of detection results								
	CPA			RT-PCR			PCR		
1	+	+	+	+	+	+	+	+	+
2	+	+	+	+	+	+	+	+	+
3	+	+	+	+	+	+	+	+	+
4	+	+	+	+	+	+	+	+	+
5	+	+	+	+	+	+	+	+	+
6	+	+	+	+	+	+	+	+	+
7	+	+	+	+	+	+	+	+	+
8	+	+	+	+	+	+	+	+	+
9	+	+	+	+	+	+	+	+	+
10									
11	+	+	+	+	+	+	+	+	+
12									
13									
14									
15	+	+	+	+	+	+	+	+	+
16	+	+	+	+	+	+	+	+	+
17	+	+	+	+	+	+	+	+	+
18									
19									
20	+	+	+	+	+	+	+	+	+
21									
22									
23									
24									
25	+	+	+	+	+	+	+	+	+
26	+	+	+	+	+	+	+	+	+
27									
28									
29									
30									
31									
32									
33	+	+	+	+	+	+			
34	+	+	+	+	+	+	+	+	+
35	+	+	+	+	+	+	+	+	+
36	+	+	+	+	+	+	+	+	+
37									
38	+	+	+	+	+	+	+	+	+
39									

40	+	+	+	+	+	+	+	+	+
41	+	+	+	+	+	+	+	+	+
42	+	+	+	+	+	+	+	+	+
43	+	+	+	+	+	+	+	+	+
44	+	+	+	+	+	+	+	+	+
45									
46									
47									
48									

"+" indicates that the ALV-J test result is positive

Supplementary Table S2 Detection results for cell cultures inoculated with clinical plasma with 0.1 ≤S/P ≤0.2

Sample	Three methods of detection results								
No.	CPA			qPCR			PCR		
1	+	+	+	+	+	+			
2									
3	+	+	+	+	+	+			
4	+	+	+	+	+	+			
5									
6	+	+	+	+	+	+	+	+	+
7	+	+	+	+	+	+	+	+	+
8									
9	+	+	+	+	+	+	+	+	+
10	+	+	+	+	+	+	+	+	+
11	+	+	+	+	+	+	+	+	+
12									
13	+	+	+	+	+	+			
14	+	+	+	+	+	+			
15									
16				+	+	+			
17	+	+	+	+	+	+	+	+	+
18	+	+	+	+	+	+			
19	+	+	+	+	+	+	+	+	+
20									
21	+	+	+	+	+	+	+	+	+
22	+	+	+	+	+	+			
23	+	+	+	+	+	+			
24									
25									
26	+	+	+	+	+	+	+	+	+
27	+	+	+	+	+	+	+	+	+
28									
29									
30									
31	+	+	+	+	+	+	+	+	+
32	+	+	+	+	+	+	+	+	+
33				+	+	+			
34									
35	+	+	+	+	+	+	+	+	+
36	+	+	+	+	+	+	+	+	+
37	+	+	+	+	+	+			
38	+	+	+	+	+	+			
39									
40	+	+	+	+	+	+	+	+	+
41									
42	+	+	+	+	+	+	+	+	+
43	+	+	+	+	+	+	+	+	+
44									
45	+	+	+	+	+	+	+	+	+
46	+	+	+	+	+	+	+	+	+

47									
48									
49	+	+	+	+	+	+	+	+	+
50									
51	+	+	+	+	+	+	+	+	+
52	+	+	+	+	+	+			
53	+	+	+	+	+	+			
54	+	+	+	+	+	+	+	+	+
55	+	+	+	+	+	+	+	+	+
56									
57	+	+	+	+	+	+	+	+	+
58									
59	+	+	+	+	+	+			
60									
61	+	+	+	+	+	+			
62									
63	+	+	+	+	+	+	+	+	+
64	+	+	+	+	+	+	+	+	+
65									
66	+	+	+	+	+	+	+	+	+
67	+	+	+	+	+	+	+	+	+
68	+	+	+	+	+	+	+	+	+
69									
70	+	+	+	+	+	+			
71									
72									
73									
74	+	+	+	+	+	+			
75									

"+" indicates that the ALV-J test result is positive
